# Nucleosome and ubiquitin position Set2 to methylate H3K36

**DOI:** 10.1038/s41467-019-11726-4

**Published:** 2019-08-22

**Authors:** Silvija Bilokapic, Mario Halic

**Affiliations:** Department of Structural Biology, St. Jude Children’s Research Hospital, 263 Danny Thomas Place, Memphis, TN 38105 USA

**Keywords:** Cryoelectron microscopy, Histone post-translational modifications

## Abstract

Histone H3 lysine 36 methylation (H3K36me) is a conserved histone modification deposited by the Set2 methyltransferases. Recent findings show that over-expression or mutation of Set2 enzymes promotes cancer progression, however, mechanisms of H3K36me are poorly understood. Set2 enzymes show spurious activity on histones and histone tails, and it is unknown how they obtain specificity to methylate H3K36 on the nucleosome. In this study, we present 3.8 Å cryo-EM structure of Set2 bound to the mimic of H2B ubiquitinated nucleosome. Our structure shows that Set2 makes extensive interactions with the H3 αN, the H3 tail, the H2A C-terminal tail and stabilizes DNA in the unwrapped conformation, which positions Set2 to specifically methylate H3K36. Moreover, we show that ubiquitin contributes to Set2 positioning on the nucleosome and stimulates the methyltransferase activity. Notably, our structure uncovers interfaces that can be targeted by small molecules for development of future cancer therapies.

## Abstract (web summary)

Set2 methyltransferase catalyzes the lysine 36 methylation of histone H3 (H3K36me) and the enzyme is mutated in many cancers. The authors provide mechanistic insights into how Set2 methylates nucleosomes by determining the 3.8 Å cryo-EM structure of Set2 bound to a H2B ubiquitinated nucleosome core particle.

## Introduction

Histone modifications are a key player in maintaining genome stability, chromosome segregation, and genome expression^1^. One of the essential histone modifications, histone H3 lysine 36 methylation (H3K36me) is deposited by the Set2 methyltransferase onto the H3 tail and is conserved from yeast to humans^2,3^. Set2 is recruited by phosphorylated C-terminal domain of RNA Polymerase 2 during transcription and catalyzes H3K36 methylation over gene bodies^4–9^. In addition to Set2, RNA Polymerase 2 elongation complex recruits Bre1/Rad6 that ubiquitinate H2B K120^10,11^, indicating possible interplay between these two histone modifications. Both, H3K36me and H2B K120 ubiquitination are enriched over gene bodies^12^. H3K36me recruits Rpd3S histone deacetylase complex to restore a repressed chromatin state behind the elongating polymerase^13,14^. Although deposited by the transcription machinery, H3K36me seems to be a silencing mark that prevents initiation of cryptic transcription within gene bodies^2,15–17^. Recent data show that Set2 and H3K36me are also involved in DNA damage repair^18–20^, regulation of pre-mRNA splicing^21,22^, chromatin condensation^23^, and histone exchange^24,25^.

In animals, Set2 family enzymes are essential for maintenance of cell identity^26,27^ and are mutated in many patients with clear cell renal cell carcinoma, acute leukemias, bladder cancer, and glioblastoma^28–30^. Recently, mutation of H3.3K36 to methionine has been found in chondroblastomas, head and neck squamous cell carcinoma, and colorectal cancer^31–36^. H3.3K36M mutation traps SETD2 methyltransferase on the substrate which results in genome-wide loss of H3K36 methylation and tumor formation^33,35^. Set2 family enzymes are overexpressed in multiple myeloma, neuroblastoma, bladder and breast cancers, and correlate with poor prognosis^3,37–40^. These findings show that overexpression or mutation of Set2 enzymes or H3K36 promote cancer progression.

Although Set2 involvement in essential cellular processes is well described, mechanisms of Set2 mediated H3K36 methylation are poorly understood. In this study, we present 3.8 Å cryo-EM structure of Set2 bound to the mimic of H2B ubiquitinated nucleosome core particle (NCP)^41^. Our structure shows that Set2 makes extensive interactions with H3 αN, H3 tail, H2A C-terminal tail, and stabilizes DNA in an unwrapped conformation^42^. These interactions with the nucleosome are required for the specificity toward H3K36 methylation. Moreover, we show that the AWS domain of Set2 interacts with ubiquitin which promotes H3K36 methylation. Our structure reveals the interface between nucleosome and Set2, which is a potential drug target for cancer treatments.

## Results

### Cryo-EM structure of Set2 bound to nucleosome

**Fig. 1:**
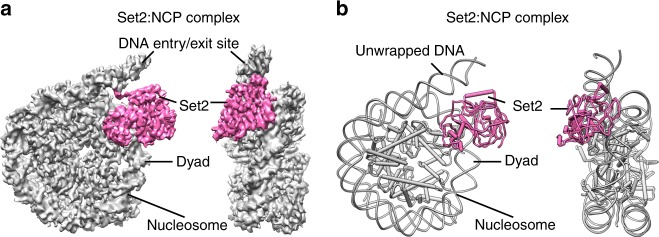
Cryo EM structure of Set2 methyltransferase bound to the NCP. **a** Cryo-EM map of Set2 bound to NCP at 3.8 Å. NCP is shown in gray and Set2 in pink. Set2 stabilizes NCP with the unwrapped DNA. **b** Model for cryo-EM map of the Set2 bound to NCP. NCP is shown in gray and Set2 in pink

Histone H3K36me3 and H2BK120 ubiquitination are hallmarks of active chromatin. To visualize the mechanism of H3K36 methylation, we assembled the complex of Set2 and the mimic of H2B ubiquitinated nucleosome^43^, the substrate of Set2 (Supplementary Fig. 1a-d). To stabilize the complex we have introduced H3K36M mutation^44,45^, recently identified in childhood cancers (Supplementary Fig. 1e). In the electron micrographs the complex is present in various orientations and in a subset of 2D class averages an additional density was bound to the NCP (Supplementary Fig. 1f, g). In initial 3D reconstruction, with overall resolution at 3.4 Å, a noisy density representing Set2 domain was present near the DNA entry/exit site of the nucleosome (Supplementary Fig. 2). ~14% of the initial particles contained Set2 and further focused classification improved the Set2 density yielding a map at 3.8 Å (Set2:NCP) (Table 1, Fig. 1a and Supplementary Fig. 2, 3a). Remarkably, not only NCP, but also Set2 is resolved to high resolution allowing us to describe interactions in molecular details. In this map, resolution of Set2 on the nucleosome proximal side is similar to the nucleosome with many side chains resolved, whereas on the nucleosome distal side the resolution drops to ~4–5 Å (Supplementary Fig. 3b-c). Although we used full-length Set2 for the complex formation, in our map we observe only densities for the AWS and the SET domains (Supplementary Fig. 3d). This is in agreement with previous data showing that the SET domain is sufficient to bind nucleosome and to methylate H3K36^6,46,47^. We refined the cryo-EM model of the nucleosome assembled on 601 DNA^42,48^ (PDB:6FQ5) and the Set2 model (PDB:5V21, 5JJY)^44,45^ and observed several changes in their structures when assembled in the complex (Fig. 1b and Supplementary Fig. 3e).

**Table 1:** Cryo-EM data collection, refinement, and validation statistics

	Set2_NCP EMD-0559 PDB ID 6NZO	Set2_Ub_NCP EMD-20517 PDB ID 6PX3	NCP EMD-20516 PDB ID 6PX1
*Data collection and processing*			
Magnification			
Voltage (kV)	300	300	300
Electron exposure (e–/Å^2^)	75	75	75
Defocus range (μm)	−0.7 to −3.0	−0.7 to −3.0	− 0.7 to −3.0
Pixel size (Å)	1.04	1.04	1.04
Symmetry imposed	C1	C1	C2
Initial particle images (no.)	∼1,500,000	∼1,500,000	∼1,500,000
Final particle images (no.)	66,000	50,000	210,000
Map resolution (Å) FSC threshold	3.8	4.1	3.3
Map resolution range (Å)	3.6–5.0	3.8–8.0	3.1–3.5
*Refinement*			
Initial model used	6FQ5	6FQ5	6FQ5
Model resolution (Å) FSC threshold	3.8	4.1	3.3
Model resolution range (Å)	235–3.8	235–4.1	235–3.3
Map sharpening *B* factor (Å^2^)	−100	0	−120
Model composition			
Nonhydrogen atoms	13740	14336	11939
Protein residues	990	1060	752
Ligands	3	3	0
*B* factors (Å^2^)			
Protein	55.16	297.17	41.58
Ligand	129.02	397.28	
R.m.s. deviations			
Bond lengths (Å)	0.007	0.008	0.006
Bond angles (°)	0.914	0.929	0.853
Validation			
MolProbity score	1.47	1.55	1.01
Clashscore	3.44	4.62	2.31
Poor rotamers (%)	0.12	0.45	0
Ramachandran plot			
Favored (%)	95.11	95.45	98.10
Allowed (%)	4.89	4.55	1.90
Disallowed (%)	0.0	0.0	0.0

**Fig. 2:**
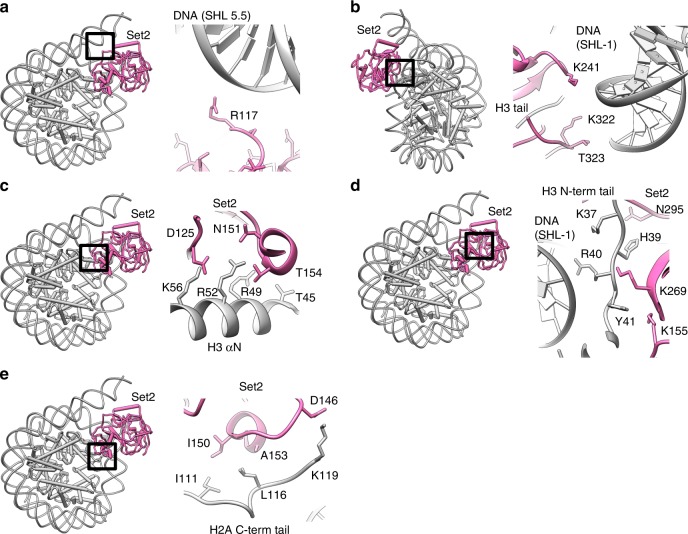
Molecular interactions between Set2 and NCP. Global location of the depicted interaction between Set2 and the nucleosome is shown on the left and close up on the right. **a** Set2 residue R117 binds the backbone of the unwrapped DNA at SHL 5.5. **b** Set2 residues K241, K322, and T323 bind the backbone of the DNA at SHL-1. **c** Set2 binds H3 αN. Set2 residues D125, N151, and T154 bind the H3 K56, R52, R49 and T45. **d** The H3 tail makes extensive interactions with Set2 and the DNA. H3 R40 and K37 bind the DNA at SHL-1. H3 Y41 and H39 interact with Set2 residues K155, K268, and N295. Set2 residue K269 interacts with the main chain of H39. **e** Set2 binds the H2A C-terminal tail. Set2 D146 makes charged interaction with H2A K119. Set2 residues I150 and A153 make hydrophobic interactions with the H2A residues I111 and L116, respectively

Our cryo-EM structure shows that Set2 stabilizes the unwrapped state of the nucleosome, which resembles the Class 2 state we have previously observed^42^ (Fig. 1a, b). The DNA unwrapping is required for Set2 to bind H3K36 that is located close to the DNA entry/exit site of the nucleosome. The conserved positively charged Set2 residue R117 binds the backbone of the unwrapped DNA at SHL 5.5 and stabilizes the open conformation (Fig. 2a and Supplementary Fig. 3e, f). Moreover, Set2 makes a set of electrostatic interactions with the backbone of the DNA near the dyad (Fig. 2b and Supplementary Fig. 3g). This interaction with the DNA at SHL-1 rearranges the Set2 Post-Set domain when compared with the crystal structure^44^ (Supplementary Fig. 3h).

In addition to the DNA, Set2 makes multiple interaction with histones H3 and H2A that position Set2 catalytic domain for functional interaction with H3K36. Set2 residues D125 and N151 interact with positively charged residues K56 and R52, while H3 R49 and T45 face toward the Set2 A153 and T154 (Fig. 2c). These H3 αN residues organize the last 13 bp of DNA in the nucleosome, but as DNA is unwrapped in the complex, the side chains adopt different conformation and bind Set2 (Supplementary Fig. 3c, i). Set2 interaction further extends to the H3 tail where H3 Y41 binds into the pocket formed by H3 residues R49, V46, and T45, and by Set2 residues K155 and K269. Y41 aromatic ring packs against invariant Set2 K269, which together with N295 and N297 also coordinates H3 H39 (Fig. 2d and Supplementary Fig. 4a). When H3 tail is bound to Set2, positively charged residues H3 R40 and K37 face toward the DNA and contribute to position the H3 tail for the interaction with Set2 (Fig. 2d). Whereas H3 M36(K36) is positioned in a similar way to Set2 crystal structures^44,45^, the H3 residues following the H3 P38 take a different path in our structure and bind both Set2 and the nucleosomal DNA (Supplementary Fig. 4b, c). It has been suggested that P38 plays a role in the H3K36 methylation^49^ and our data show that the H3 tail kinks at the P38 to accommodate restrains provided by the nucleosome and to allow for Set2 binding.

**Fig. 3:**
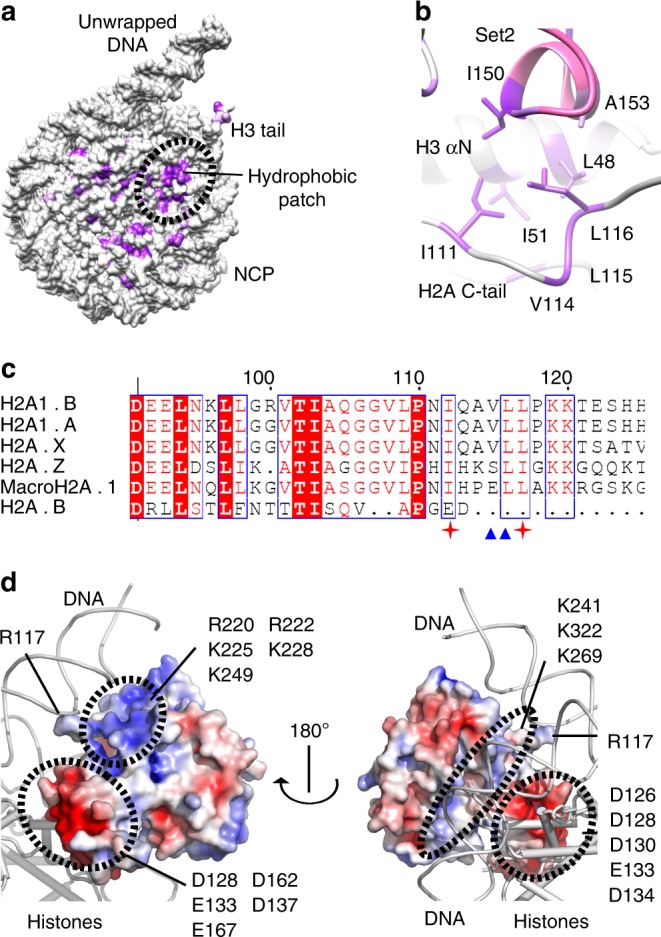
Set2 makes hydrophobic and charged interactions with NCP. **a** Hydrophobic residues on the surface of NCP are shown in violet. Black dashed circle marks hydrophobic patch on the NCP. **b** Hydrophobic patch of NCP is formed by H3 αN residues I51 and L48 and H2A C-terminal tail residues I111, L115, and L116. Set2 residues I150 and A153 bind the hydrophobic patch of NCP. **c** Sequence alignment of the C-terminal tail of the representative H2A histone variants. Conserved residues are highlighted in red. The position of I111 and L116, residues that build hydrophobic pocket on the nucleosomal surface, are labeled with the red cross. V114 and L115 that position H2A C-terminal tail are marked with blue triangle. Residues important for the interactions with Set2 enzyme are not conserved in H2A.B. The alignment was generated with the ESPript server (http://espript.ibcp.fr)^83^. **d** Electrostatic surface potential of Set2. Set2 surface facing DNA is predominately positively charged (blue), whereas Set2 residues facing positively charged histones are mainly negatively charged (red)

Beside the interactions with H3, Set2 residue D146 makes an interaction with K119 in the H2A C-terminal tail and rearranges the tail (Fig. 2e and Supplementary Fig. 4d). Moreover, Set2 residues I150 and A153 make hydrophobic interactions with the H2A C-terminal tail I111 and L116, and H3 αN residues I51 and L48 (Fig. 2e). In addition to prevailing charged interactions, our structure shows that Set2 binds hydrophobic patch of the nucleosome formed by the H2A C-terminal tail and the H3 αN (Fig. 3a, b and Supplementary Fig. 4e). This hydrophobic patch, utilized by Set2 is likely to be another hot spot for interactions with the nucleosome, similarly to the acidic patch^50^. Biochemical data have shown that Sgo1, COC complex, AKT protein kinase B, ISWI remodelers, and many other complexes require residues in the hydrophobic patch for nucleosome binding and the activity^51–53^ and might bind the nucleosome in a similar way to Set2. Notably, we do not observe Set2 SET domain binding to the acidic patch.

We observed that histone H2A variant H2A.B does not have the hydrophobic residues that interact with Set2 (Fig. 3c and Supplementary Fig. 5a, b). H2A.B histone variant is associated with active transcription^54,55^, however, in contrast to the canonical H2A, in vivo data show that H2A.B containing nucleosomes are not methylated on H3K36^55^. Residues essential for Set2 binding are not present in H2A.B nucleosomes and our results explain why these nucleosomes do not have H3K36me, although found in regions that are methylated on H3K36. Our data provide insight into the modulation of chromatin landscape by interplay between histone post-translational modifications and histone variants.

In our structure Set2 makes extensive charged and hydrophobic interactions with the DNA and the histones. The structure shows that Set2 residues facing the DNA are predominately positively charged, whereas, the residues that are facing the positively charged histones are negatively charged (Fig. 3d). Notably, the residues interacting with the nucleosome show higher degree of conservation (Supplementary Fig. 5c).

### H2BK120 ubiquitination stimulates Set2 activity

**Fig. 4:**
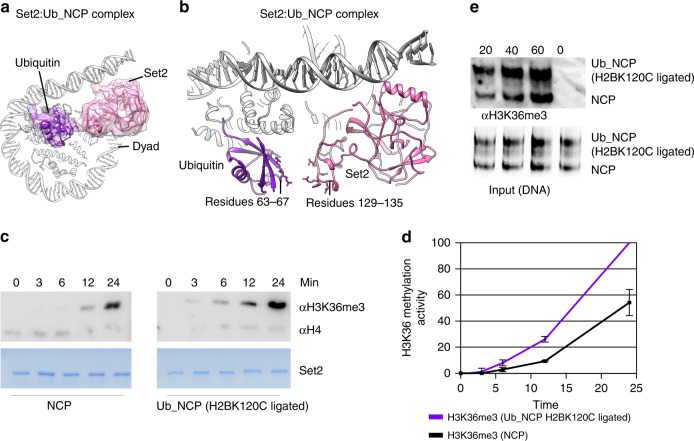
Ubiquitin binds Set2 and stimulates Set2 H3K36me3 activity. **a** Cryo-EM map resolved to 4.1 Å showing a subset of particles with defined density for ubiquitin, which interacts with Set2. Ubiquitin density and model are shown in violet. Set2 density and model are shown in pink. NCP is shown as model in gray. **b** The model showing overall interaction between Set2 and ubiquitin. Ubiquitin residues 63–67 come to close proximity to Set2 AWS domain (residues 129–135). **c** Set2 methyltransferase assay with the unmodified and the H2BK120C ubiquitinated nucleosomes. In presence of H2BK120C ubiquitin, Set2 shows increased H3K36me3 when compared with unmodified nucleosome. **d** Quantification of Set2 methyltransferase activity assay from (**c**) . Error bars show standard error of three independent replicates. **e** Set2 methyltransferase competition assay with equimolar amounts of both the unmodified and the H2BK120C ubiquitinated nucleosomes. The nucleosomes were separated on the native gel: sample stained with SybrGold is shown in the lower panel and tri-methylation activity detected with H3K36me3 antibody in the upper panel. Upper band is the H2BK120C ubiquitinated nucleosomes and the lower band are the unmodified nucleosomes. Source data are provided as a Source Data file

Set2 is recruited to chromatin by RNA Polymerase II elongation complex, which also recruits Bre1 E3 ligase that ubiquitinates H2B. Moreover, recent in vivo data reveal a link between H2B ubiquitination and H3K36me^41^. Therefore, we have postulated that H2B ubiquitination might stabilize the Set2:NCP complex for cryo-EM studies. In the Set2 map, the density for ubiquitin is visible only at low contour level, indicating local flexibility (Supplementary Fig. 6a, b). We have used focused classification to improve the ubiquitin density, which yielded maps with no visible ubiquitin, flexible ubiquitin, and defined ubiquitin (Supplementary Fig. 6c). In all maps, Set2 was in the same conformation, indicating that H3K36M mutation is the primarily stabilizing factor in our construct. In the cryo-EM map (Set2:Ub_NCP) at 4.1 Å ubiquitin is resolved to 5–8 Å and secondary structure elements are visible permitting us to fit the ubiquitin crystal structure (PDB:1UBQ) (Fig. 4a and Supplementary Fig. 6d-g). In our complex, Set2 residues 129–135 of the AWS domain come to proximity to residues 63–67 of the C-terminal β-strand of ubiquitin (Fig. 4b and Supplementary Fig. 6e).

Although this interaction is not well defined in the structure, our data suggest that ubiquitin helps positioning Set2 on the nucleosome. Therefore, we have generated ubiquitinated nucleosomes by ligating ubiquitin to H2B K120^56^ (Supplementary Fig. 7a-f) and by fusing ubiquitin to the H2A N-terminus (R17), which is an efficient mimic of naturally occurring H2BK120 ubiquitination^43,57^ (Supplementary Fig. 1b-d and Supplementary Fig. 7g). Our data show that Set2 activity is higher on both mimics of H2B K120 ubiquitinated nucleosomes when compared with the unmodified nucleosomes, indicating that ubiquitin stimulates the activity (Fig. 4c, d and Supplementary Fig. 8a, b). Further, we added Set2 to an equimolar mix of unmodified and H2B ubiquitinated nucleosomes, and observed that Set2 preferably methylates H2B ubiquitinated nucleosomes (Fig. 4e). Although H2B K120 ubiquitin increases Set2 activity, it does not increase Set2 binding to the nucleosome, suggesting that ubiquitin rather promotes positioning of the Set2 active site over the substrate (Supplementary Fig. 8c). In conclusion, our data show that H2B K120 ubiquitination facilitates H3K36me3 activity of Set2.

### Auxiliary domains of Set2 bind nucleosome

**Fig. 5:**
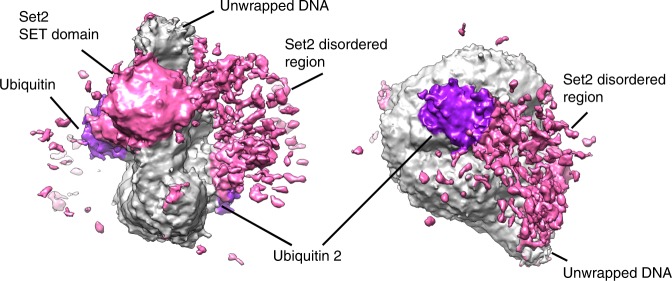
Set2 auxiliary regions bind the nucleosome. Cryo-EM map resolved to 4.2 Å showing a subset of particles with the noisy density next to the nucleosome. The noisy density is spanning from the Set2 SET domain and the unwrapped DNA to the second ubiquitin on the opposite side of the nucleosome. This density is generated by auxiliary Set2 domains and disordered regions. Ubiquitin is shown in violet, Set2 in pink, and NCP in gray

Although two Set2 could bind one nucleosome, we did not find a single class with the two Set2 bound. We have, however, observed a cluster of a noisy density on the side of the nucleosome opposite to the bound Set2 SET domain (Fig. 5). Further unfocused classification has enriched for this noisy density generated by the remaining Set2 sequence, which includes several domains and disordered regions (Supplementary Figs. 3d, 9a-b). This less defined parts of Set2 interact with the Set2 SET domain, unwrapped DNA, ubiquitin and the histone core at multiple sites (Fig. 5). Our data reveal that the auxiliary domains and disordered regions of Set2 bind the nucleosome and ubiquitin, and this prevents binding of the second Set2 to the nucleosome (Fig. 5).

## Discussion

In conclusion, our data show that Set2 interactions with the H3 αN and the H2A C-terminal tail position Set2 on the nucleosome to specifically bind and methylate H3K36. In agreement with our structure, previous work has shown that hydrophobic residues I111, L115, and L116 in the H2A C-terminal tail are required for the H3K36 methylation and repression of cryptic transcription^58,59^. Consistently, mutations in H2A residues residues Q114 and N115, that do not bind Set2 in our structure, showed no or little effect on H3K36me^58^. Notably, ubiquitination of H2AK119 was shown to inhibit Set2 activity, which is consistent with our structure in which Set2 makes extensive interactions with this part of the H2A tail^60^.

Mutational analysis has shown that residues T45, R49, and R52 in H3 αN are required for H3K36 methylation and repression of cryptic transcription^58,59,61,62^, in agreement with our structure. Previous work has shown that H4K44 is required for Set2 activity^58,61^. Although we do not see direct interaction of H4K44 with the Set2 SET domain, our data show that H4K44 organizes the H2A C-terminal tail and the hydrophobic patch that are essential for Set2 activity.

The Set2:nucleosome complex is further stabilized by the interaction with the DNA, which is consistent with observations that DNA is required for Set2 activity^61,63^. Moreover, in absence of the intact nucleosome Set2 family enzymes show spurious activity toward H3K4, H3K27, H4K20, H4K44, H2A, and H2B residues^3,63^. Our structures explain how interaction with the nucleosome provides the specificity to H3K36 methylation.

Our data show that the Set2 AWS domain interacts with the ubiquitin on H2BK120, suggesting that ubiquitin helps positioning Set2 on the nucleosome. This is supported by our biochemical work that shows increased Set2 tri-methylation activity on H2BK120 ubiquitinated nucleosomes, when compared with the unmodified nucleosomes. Moreover, it is consistent with the in vivo data showing reduced H3K36me3 in deletion of the Bre1 E3 ligase^43^, which is required for H2B K120 ubiquitination. Previous biochemical and in vivo data also noted the considerable spatial plasticity in the localization of the residue that is ubiquitinated for nucleosome methylation by Dot1 enzyme. Ubiquitin ligated to H2BK120, H2BK125, H2AK22, and fused to H2AS17 stimulated Dot1 activity^43,57,64^. In agreement, our data show that ubiquitin ligated to H2BK120 and fused to H2AR19 stimulates Set2 activity. Since ubiquitin is linked to the nucleosome by intrinsically flexible C-terminal tail, it can adopt many positions on the nucleosome. Remarkably, the position of ubiquitin on the nucleosome surface is unique in each of several recently published structures of various factors bound to the H2BK120 ubiquitinated nucleosomes^65–67^ (Supplementary Fig. 9c). This indicates that binding to the interacting partner determines position of the flexible ubiquitin on the nucleosome.

Recently, a structure of H3K36 methyltransferase ASH1L was determined in a complex with its activation partner MRG15^68,69^. Superposition of the SET domains from ASH1L:MRG15 complexes (PDB ID: 6ago, 6ine) and Set2:NCP complex shows that different interfaces of Set2 bind nucleosome and MRG5 (Supplementary Fig. 9d). This indicates that an activating factor MRG5 can bind Set2 that is bound to the nucleosome to further stimulate its activity.

In our structure we observed a cluster of less defined density that spans from the SET domain and unwrapped DNA to the H2A/H2B on the opposite side of the nucleosome. Our structure suggest that remaining Set2 domains and/or disordered density bind the SET domain, ubiquitin, unwrapped DNA, and histone core at multiple sites. This is in agreement with biochemical data showing that full-length Set2 shows higher activity on nucleosomes compared with the isolated SET domain^70^, indicating that auxiliary domains promote and modulate H3K36 methylation.

Perturbations in the enzymes that maintain the levels of H3K36 methylation have been reported in many diseases, such as Sotos syndrome, acute myeloid leukemia, multiple myeloma, and prostate cancer. Set2 enzyme NSD2 is overexpressed in multiple myeloma, bladder, and several other cancers, which correlates with poor prognosis^37,38,71^, making these enzymes potential drug targets. The interaction with the nucleosome we describe in this study could be targeted by small molecules to inhibit Set2 enzymes in treatment of various cancers.

## Methods

### Chaetomium thermophilum genomic DNA preparation

Twenty milliliters of saturated *C. thermophilum* culture were harvested by centrifugation. Cells were resuspended in 0.5 ml of dH_2_O and flash frozen as small droplets in liquid nitrogen. Cells were pulverized under dry ice by mortar and pestle, thawed, and resuspended in extraction buffer (2 ml of 2% Triton X-100, 1% SDS, 100 mM NaCl, 10 mM Tris/HCl, pH 8.0, 1 mM EDTA). In total, 1.5 ml of phenol:chloroform:isoamyl alcohol (25:24:1) and glass beads were added to pulverized cells. The mixture was vigorously mixed by vortex for 5 min and aqueous layer was isolated by centrifugation. DNA was precipitated using sodium acetate (final concentration 0.3 M, pH 5.2) and 2.5 volumes of ice-cold ethanol, collected by centrifugation and redissolved in TE buffer (10 mM Tris/HCl, pH 8.0, 1 mM EDTA).

### Plasmid construction

*Chaetomium thermophilum* (source Ed Hurt lab) Set2 was amplified from the genomic DNA and cloned into a pET-Duet plasmid to yield an N-terminally 6xHis-tagged protein followed by a human rhino-virus 3C protease recognition site. Two intrones contained within the genomic DNA were removed by iPCR^72^. Oligonucleotides used for cloning are listed in Supplementary Table 1. The resulting vector, listed in Supplementary Table 2, was transformed into LOBSTR-BL21(DE3)-RIL (Kerafast)^73^.

*Xenopus laevis* histone H3K36M and H2BK120C mutant plasmids were generated by iPCR using oligonucleotides listed in Supplementary Table 1. The resulting vector was transformed into BL21(DE3)-pLysS bacterial cell.

Ubiquitin fused to the N-terminus of H2A is an efficient mimic of naturally occurring H2BK120 ubiquitination^43^. Gene coding for *S. cerevisiae* ubiquitin was PCR amplified from genomic DNA and cloned into NcoI recognition sequence in the plasmid for *X. laevis* coexpression of soluble H2A/H2B histone dimer^43^. Restriction enzyme cloning positioned ubiquitin gene between region coding for 6xHis-tag and H2A. A short HA tag used for tethering ubiquitin to H2A^43^ was replaced in our construct with an equally long Gly-Ser linker by iPCR. The final plasmid construct contained N-terminal 6xHis-tag followed by thrombin recognition site, ubiquitin and nine residue Gly-Ser linker tethered to H2A R17. Oligonucleotides used are listed in Supplementary Table 1.

### Inverse PCR

iPCR reactions were setup in a total volume of 25 μl. Products were analyzed on the agarose gel and purified using the NucleoSpin Gel and PCR Clean-up kit. Ten microliters of purified PCR product were incubated with 1 × T4 DNA ligase buffer and 5 U of T4 PNK for 1 h at 37 °C in a total volume of 20 μl. T4 DNA ligase (200 U) was added to the reaction and incubated for 1 h at room temperature. After addition of 10 U of DpnI, the reaction was incubated for 1 h at 37 °C and 5 μl were transformed into competent XL1-Blue *E. coli* cells. All obtained plasmid sequences were checked by DNA sequencing.

### Set2 expression and purification

*E. coli* Rosetta (Novagen) bacterial culture, containing vector for Set2 overexpression, was grown in LB medium containing the appropriate antibiotics and 0.4% (w/v) glucose at 37 °C. When the optical density reached 0.6 at 600 nm, the culture was shifted to 18 °C for 30 min before it was induced by the addition of 0.2 mM IPTG. After overnight induction at 18 °C, cells were harvested by centrifugation, resuspended in lysis buffer (50 mM sodium-phosphate, pH 8.0, 500 mM NaCl, 20 mM imidazole, 3 mM β-mercaptoethanol, 1 mM phenylmethanesulfonyl fluoride and Benzonase) and lysed using a french press. The cleared supernatant was incubated with Ni Sepharose 6 Fast Flow resin for 30 min at 4 °C. The resin was extensively washed with lysis buffer in batch and loaded onto a disposable column. On the column, the resin was washed with 4 bed volumes of washing buffer (50 mM sodium-phosphate, pH 8.0, 500 mM NaCl, 40 mM imidazole, 3 mM β-mercaptoethanol) and the bound protein was eluted with 4 bed volumes of elution buffer (50 mM sodium-phosphate, pH 8.0, 500 mM NaCl, 300 mM imidazole, 3 mM β-mercaptoethanol). The protein was dialyzed overnight at 4 °C against 20 mM HEPES/NaOH, pH 7.5, 200 mM NaCl, 0.1 mM EDTA, 1 mM DTT. The N-terminal tag was proteolytically removed during dialysis. The protein was further purified on size exclusion Superdex 200 Increase 10/300 equilibrated in 20 mM HEPES/NaOH, pH 7.5, 200 mM NaCl, 1 mM DTT. All protein purification step were done at 4 °C. The fractions with the protein were concentrated, flash frozen and stored at −80 °C as single use aliquots (Supplementary Fig. 1a).

### Histone expression and purification

The plasmid for coexpression and copurification of *X. laevis* histone pairs (6xHis_HRV3C_H2A/H2B, 6xHis_Ubq_H2A/H2B, 6xHis_HRV3C_H3/H4) were transformed into *E. coli* Rosetta cells (Supplementary Table 2). Protein expressions and Ni-NTA purifications were done as described for Set2 methyltransferase enzyme except that all buffers contained 2 M NaCl. After the elution from the resin, the histone pairs were dialyzed overnight at 4 °C against 25 mM HEPES/NaOH, pH 7.5, 2 M NaCl, 0.1 mM EDTA, 1 mM DTT. The samples were further purified via cation-exchange chromatography in batch (SP Sepharose Fast Flow resin). To facilitate binding onto the resin, the samples were diluted with buffer containing 25 mM HEPES/NaOH, pH 7.5 and 1 mM DTT to 500 mM salt concentration. The resin was extensively washed with 500 mM salt buffer in batch and loaded onto a disposable column. On the column, the resin was washed with 2 bed volumes of buffer containing 700 mM salt (25 mM HEPES/NaOH, pH 7.5, 700 mM NaCl and 1 mM DTT). The samples were eluted with 4 bed volumes of 25 mM HEPES/NaOH, pH 7.5, 2 M NaCl, 1 mM DTT. Histone proteins were concentrated and further purified on the size exclusion Superdex 200 Increase 10/300 GL column equilibrated in 25 mM HEPES/NaOH, pH 7.5, 2 M NaCl, 1 mM DTT. The fractions with the protein were analyzed on SDS-PAGE, concetrated and flash frozen.

H3K36M and H3K120C mutants were overexpressed in BL21(DE3) pLysS cell strain and purified from the inclusion bodies^74^. Pelleted cells were resuspended in wash buffer (50 mM Tris-HCl, pH 7.5, 100 mM NaCl, 1 mM EDTA, 1 mM DTT) and sonicated. After centrifugation for 20 min at 4 °C and 17,000 rpm the pellet was resuspended in wash buffer with 1% (v/v) Triton X-100, sonicated once more and spun down. The pellet containing inclusion bodies of the corresponding histone protein was washed by completely resuspending it once in buffer with Triton X-100 and twice in buffer without Triton X-100. After each resuspension, the sample was spun for 20 min at 4 °C and 17,000 rpm. H2BK120C histone was further purified on cation-exchange chromatography under denaturing conditions. Shortly, the histone protein was disolved in 7 M urea, 50 mM Tris-Hcl, 400 mM NaCl, 1 mM DTT. Any insoluble components were removed by centrifugation. The protein was bound to cation-exchange column and eluted with the increasing salt gradient. After elution from the ion exchange column, the protein was extensively dialyzed against water, lyophilized and used in cross-linking reaction with ubiquitin. H3K36M was refolded with H4, also purified from the inclusion bodies, into histone tetramer. Tetramer was further purified under native conditions on ion exchange and size exclusion chromatography using protocol described above for co-expressed soluble histone pairs.

To obtain the histone octamer, we mixed individually purified H2A/H2B histone dimer (H2A/H2B, Ubq_H2A/H2B or H2A/H2BK120C_Ubq) and (H3/H4)2 histone tetramer ((H3/H4)2 or (H3K36M/H4)2) in 25 mM HEPES/NaOH, pH 7.5, 2 M NaCl, 1 mM DTT. After an overnight incubation, the histone octamer was purified from the unincorporated components on the size exclusion Superdex 200 Increase 10/300 GL column equilibrated in 25 mM HEPES/NaOH, pH 7.5, 2 M NaCl, 1 mM DTT buffer.

All protein purification steps were done at +4 °C. The protein samples were analyzed by 17% SDS-PAGE after each purification step (Supplementary Fig. 1b).

### Generation of nonhydrolyzable ubiquitin-H2B histone mimic

The expression plasmid containing a 6xHis-tag at the N-terminus of ubiquitin was a generous gift of Yao T^56^. Ubiquitin expression was induced in *E. coli* Rosetta cells with 1 mM IPTG at 37 °C for 3 h. Cell lysis and Ni-NTA affinity purification under denaturating conditions were performed as described for Set2 methyltransferase enzyme except that all buffers contained additional 6 M urea. Eluated protein was exhaustively dialyzed against four changes of water before lyophilization.

Lyophilized His6-Ub(G76C) and H2B(K120C) were individually resuspended in 100 mM sodium tetraborate, pH 8.5, 6 M urea for 1 h at room temperature. Solubilized proteins were clarified by centrifugation for 20 min at 17,000 rpm. TCEP was added to the protein samples at 10 mM final concentration and incubated at room temperature for 60 min. To remove any free reducing agent, proteins were desalted on PD10 column equlibrated in 50 mM sodium tetraborate, pH 8.5, 6 M urea. Ubiquitin and H2B-K120C histone, concentrated to ~10 mg/ml, were mixed in molar ratio of 2:1 and cross-linked with 1,3-dichloroacetone in *N,N*′-dimethylformamide for 60 min on ice^56^. The reaction was stopped with the addition of 5 mM DTT and incubated for at least 30 min on ice (Supplementary Fig. 7a).

The cross-linking reaction was dialyzed in Ni-NTA denaturing binding buffer (50 mM sodium-phosphate, pH 8.0, 500 mM NaCl, 6 M urea, 10 mM imidazole, 3 mM β-mercaptoethanol) and incubated with Ni Sepharose 6 Fast Flow resin at 4 °C for 30 min. The resin was extensively washed with the binding buffer and 4 column volumes of the washing buffer with an increased imidazole (50 mM sodium-phosphate, pH 8.0, 500 mM NaCl, 6 M urea, 20 mM imidazole, 3 mM β-mercaptoethanol). The proteins (his-tagged ubiquitin, his-tagged ubiquitin-ubiquitin, his-tagged ubiquitin-H2B) were eluted with the buffer containing 300 mM imidazole (50 mM sodium-phosphate, pH 8.0, 500 mM NaCl, 6 M urea, 300 mM imidazole, 3 mM β-mercaptoethanol). The eluted protein mixture was directly used in the subsequent refolding steps with H2A histone (Supplementary Fig. 7b).

### Reconstitution of histone dimers with H2BK120C mimic

H2A histone, for dimer reconstitution, was overexpressed in BL21(DE3) pLysS cell strain and purified from the inclusion bodies^74,75^. To remove minor impurities, H2A histone was further purified on cation-exchange chromatography under denaturating conditions as described. The purified H2A was dialyzed thoroughly against at least four changes of distilled water at 4 °C and lyophilized. Lyophilized protein was resuspended in unfolding buffer (6 M guanidinium hydrochloride, 20 mM Tris, pH 7.5, 1 mM DTT), allowed to unfold for 60 min at room temperature and spun by centrifugation for 20 min at 17,000 rpm at 4 °C.

The nickel affinity purification eluate of nonhydrolyzable ubiquitin-histone contains a mixture of ubiquitin-containing species: his-tagged ubiquitin, his-tagged ubiquitin-ubiquitin, and his-tagged ubiguitin-H2B. Thus, the ratio of H2A and ubiquitin-H2BK120C histone mimic for dimer reconstitution was estimated by SDS-PAGE and staining with SimplyBlue SafeStain. Histones were mixed at equal molar ratio, dialyzed against two changes of refolding buffer (25 mM HEPES/NaOH, pH 7.5, 2 M NaCl, 1 mM DTT) at 4 °C. Histone dimer was concentrated and further purified on a size exclusion chromatography Superdex 200 Increase 10/30 column, pre-equilibrated in buffer containing 15 mM HEPES/NaOH pH 7.5, 2 M NaCl, 1 mM DTT. The cleanest fractions were pooled, concentrated and used for histone octamer assembly (Supplementary Fig. 7c-f).

### Nucleosome reconstitution

Nucleosomes were reconstituted using the purified octamers and 149 bp DNA fragment containing the 601 nucleosome strong positioning sequence^42,48^. DNA for nucleosome reconstitution was PCR amplified from a plasmid containing the 601 DNA sequence using oligonucleotides listed in Supplementary Table 1. After ethanol precipitation, the DNA was resuspended in 15 mM HEPES-NaOH pH 7.5, 2 M NaCl, 1 mM DTT.

The nucleosome reconstitution was done by ‘double bag’ dialysis method^48^. The dialysis buttons, containing 0.25 ml of histone octamer:DNA mixture, were placed inside a dialysis bag filled with 50 ml 2 M salt buffer (15 mM HEPES-NaOH pH 7.5, 2 M NaCl, 1 mM DTT). The dialysis bag was dialyzed overnight against 1 l of 1 M salt buffer (15 mM HEPES-NaOH pH 7.5, 1 M NaCl, 1 mM DTT). The next day the dialysis bag was dialyzed for 5–6 h against 1 l buffer containing 15 mM HEPES-NaOH pH 7.5, 50 mM NaCl, 1 mM DTT. Finally, only the dialysis buttons were dialyzed for 1 h in fresh 50 mM salt buffer. All nucleosome reconstitution steps were done at +4 °C. The reconstitution results were analyzed on 6% native PAGE run in 1X TBE buffer at 200 V in the cold room (Supplementary Fig 1c). The gels were stained with SYBR gold (Thermo Fisher Scientific).

### CryoEM grid preparation and data collection

The formation of Set2:NCP complex was monitored by native PAGE (Supplementary Fig. 1d). NCPs and Set2 were incubated for 1 h on ice in 25 mM HEPES, pH 7.5, 50 mM NaCl, 1 mM DTT, 160 μm SAM. Twofold excess of Set over NCPs lead to 50% shift of the NCP band. At higher, fivefold excess of Set2 over NCPs we have observed the sample precipitation. Therefore, for cryo-EM sample preparation we have incubated NCPs with twofold excess of Set using identical conditions as for native PAGE analysis. Three microliters of the sample (1–1.2 mg/ml) were applied to freshly glow-discharged Quantifoil R2/1 holey carbon grid. After 3 s blotting time, grids were plunge-frozen in the liquid ethane using FEI Vitrobot automatic plunge freezer. Humidity in the chamber was kept at 95%.

Electron micrographs were recorded on FEI Titan Krios at 300 kV with a Gatan Summit K2 electron detector (~3350 micrographs) (Necen, Leiden, Netherlands). Image pixel size was 1.04 Å per pixel on the object scale. Data were collected in a defocus range of 7000–30,000 Å with a total exposure of 75 e/Å^2^. Fifty frames were collected and aligned with the Unblur software package using a dose filter^76^.

Several thousand particles were manually picked and carefully cleaned in Relion to remove inconsistent particles. The resulting useful particles were then used for semi-automatic and automatic particle picking in Relion. The contrast transfer function parameters were determined using CTFFIND4^77^. The 2D class averages were generated with Relion software package^78^. Inconsistent class averages were removed from further data analysis. The 3D refinements and classifications were subsequently done in Relion. All final refinements were done in Relion using the auto refine option. The initial reference was filtered to 60 Å in Relion. C1 symmetry was applied during refinements for all classes. Particles were split into two datasets and refined independently and the resolution was determined using the 0.143 cut-off (Relion auto refine option). Local resolution was determined with Relion. All maps were filtered to local resolution using Relion with a B-factor determined by Relion.

Molecular models were built using Coot^79^ and refined in Phenix^80^. Electrostatic surface potential calculation and figure was done using Pymol (The Pymol Molecular Graphics System, Version 2.0 Schrodinger, LCC)^81^. Visualization of all cryo-EM maps was done with Chimera^82^.

### Histone methyltransferase assay

In vitro histone methyltransferase assays (HMT) were performed using 0.5 μg of recombinantly purified *C. thermophilum* Set2 and 2 μg of the nucleosomal substrates assembled with 149 bp DNA along with 160 μm SAM, 25 mM Tris/HCl pH 8.4, 50 mM NaCl, 1 mM DTT. For the enzymatic reactions HMT buffer was used in the last dialysis step of the nucleosomes assembly reaction. Set2 enzyme was 20 times diluted in HMT buffer to 0.5 μg/μl (25 mM Tris/HCl pH 8.4, 50 mM NaCl, 1 mM DTT). The reaction was incubated at 40 °C and at each time point 20 μL aliquots were withdraw and the reaction stopped by adding 4xSDS loading dye. The reaction mixtures were analyzed by SDS-PAGE followed by western blot. Antibodies used include H3K36me1 (ab9048), H3K36me3 (ab9050), H3 (ab1791), and HRP-conjugated anti-rabbit secondary antibody (Biorad, 170-6515). Membrane was incubated with the primary and the secondary antibody for 1 h at room temperature with the washing steps between each incubation. The signal was visualized with chemoluminescent substrate.

For the H3K36me3 competition assay, the wild-type and H2BK120C ubiquitinated nucleosomes were mixed in one to one ratio and incubated with Set2 enzyme for 1 h at 40 °C. Four percent of glycerol with bromophenol blue (final volume) was added to the reaction and the samples were loaded onto two native PAGE gels in parallel. Ten percent of the reaction, run on the native PAGE, was stained with SYBRGold. Remaining sample was used to determine the level of the tri-methylation with H3K36me3 antibodies.

### Reporting summary

Further information on research design is available in the Nature Research Reporting Summary linked to this article.

## Supplementary information

Supplementary Information

Peer Review File

Reporting Summary

Source Data

Source Data

## Data Availability

EM densities have been deposited in the Electron Microscopy Data Bank under accession codes EMD-0559, EMD-20517 and EMD-20516. The coordinates of EM-based models have been deposited in the Protein Data Bank under accession codes PDB 6NZO, 6PX3, and 6PX1. The source data underlying Fig. 4c–e and Supplementary Figs 1a–f, 7a-fs8a-c are provided as a Source Data file. All other data are available from the corresponding author upon reasonable request.
