# Supplementary material for: Nucleosome and ubiquitin position Set2 to methylate H3K36
Source: Nat Commun. 2019 Aug 22;10:3795. doi: 10.1038/s41467-019-11726-4 (PMC6706414; doi:10.1038/s41467-019-11726-4)
Supplement: Supplementary file 1 — Supplementary Information [file 41467_2019_11726_MOESM1_ESM.pdf]

Supplementary information

Nucleosome and ubiquitin position Set2 to methylate H3K36

Silvija Bilokapic and Mario Halic

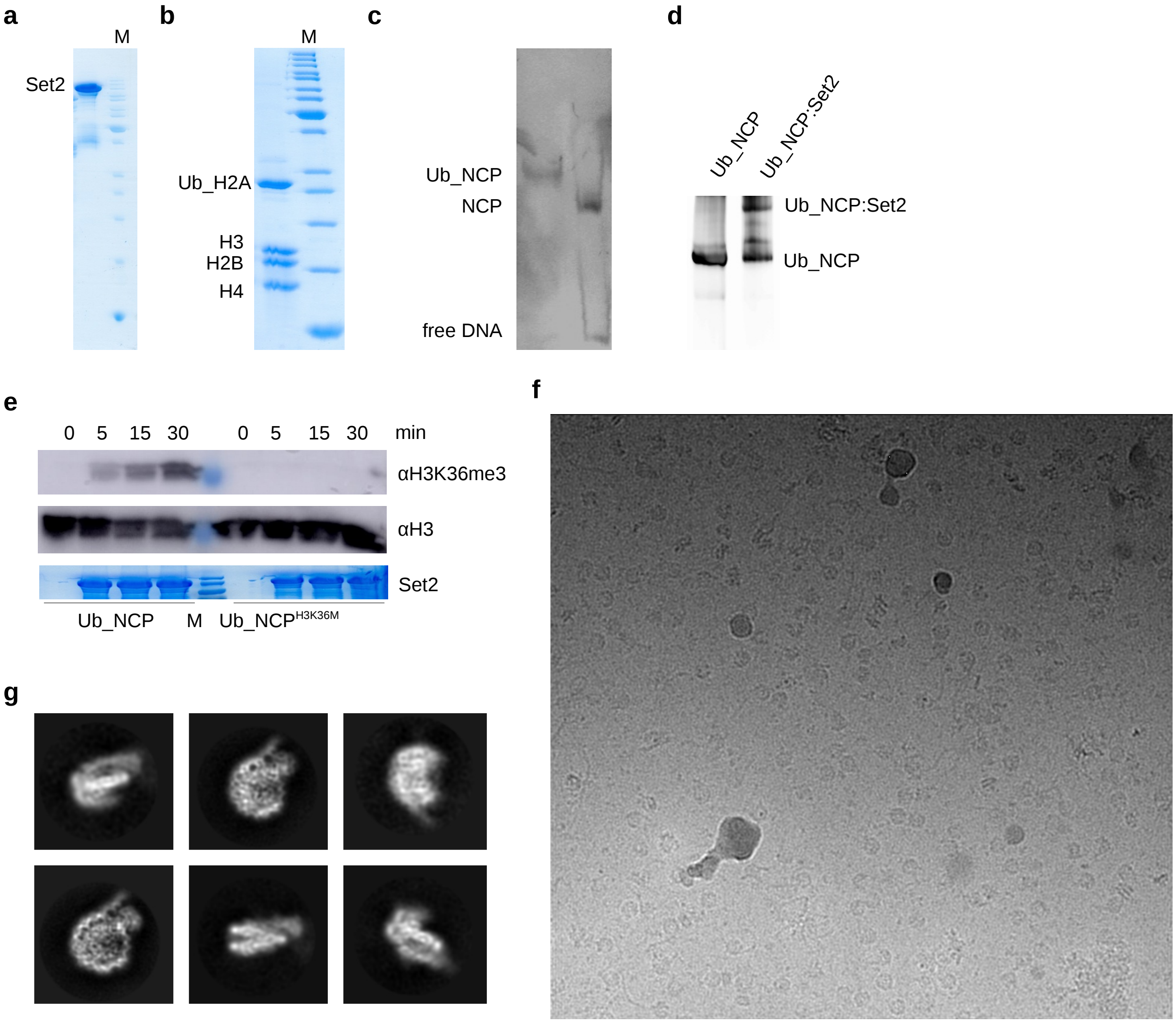

Supplementary Figure 1

Supplementary Fig. 1. Assembly and cryo-EM of the Set2:NCP complex

a) SDS-PAGE showing Set2 purification.

b) SDS-PAGE showing nucleosome assembly with H2A fused to ubiquitin.

c) Native gel showing the nucleosome assembly on 149 bp 601 DNA.

d) Native gel showing Set2:NCP complex assembly.

e) Set2 methyltransferase assay showing H3K36 methylation on Ub_NCPs. H3K36M mutation abolishes methylation.

f) Representative cryo-EM micrograph collected with Titan Krios electron microscope at 300 keV. NCPs in multiple orientations are clearly visible.

g) Representative 2D class averages showing the NCPs and Set2 bound NCPs in different orientations (side, tilted and top views). Many details are visible in 2D class averages.

Source data are provided as a Source Data file.

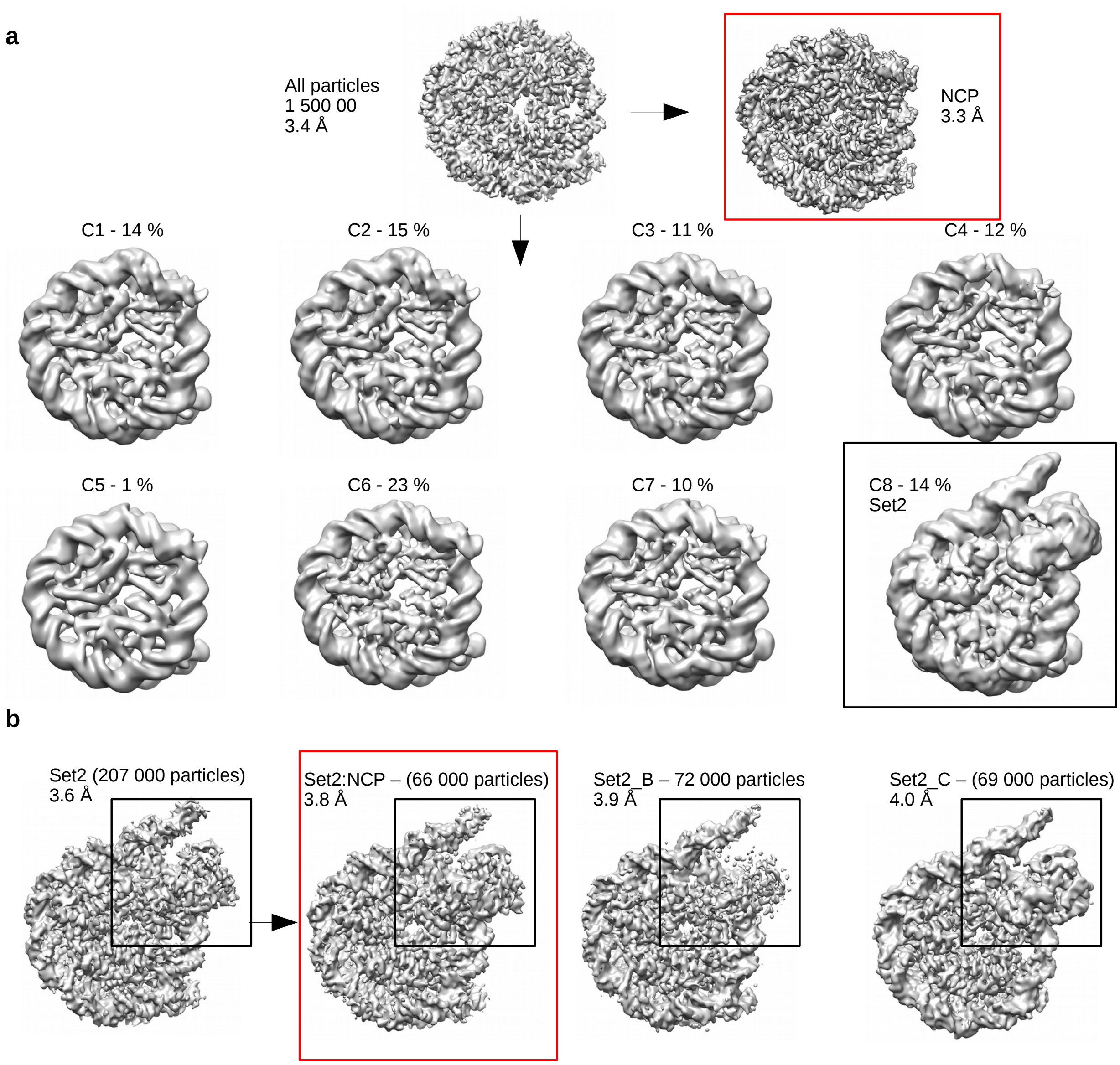

Supplementary Figure 2

Supplementary Fig. 2. Classification of the Set2:NCP complex

The overall dataset comprising 1 500 000 particles was extensively 3D classified.

a) Empty nucleosome was refined to 3.3 Å.

b) Set2 class was further classified and refined to 3.8 Å.

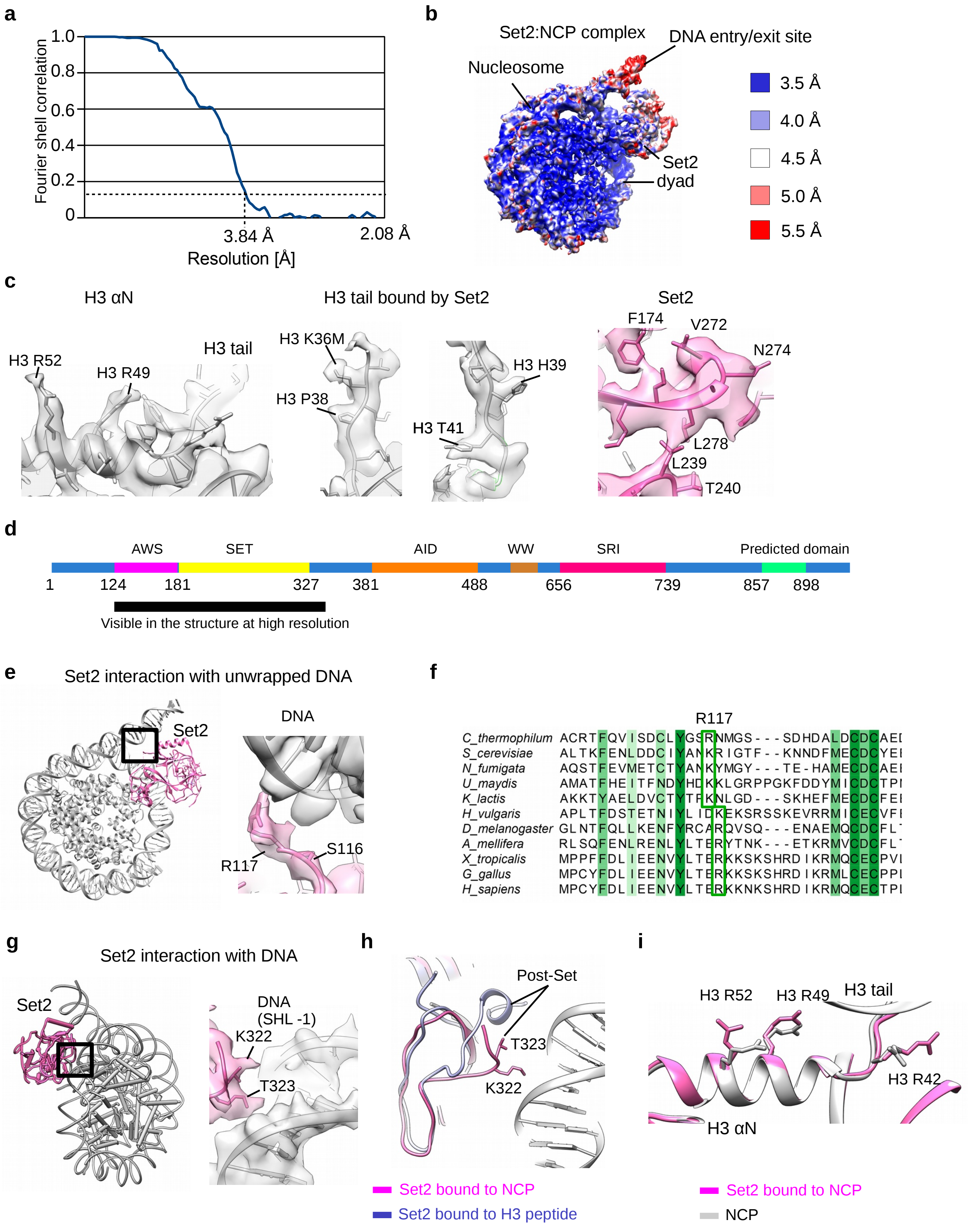

Supplementary Figure 3

Supplementary Fig. 3. Cryo-EM structure of the Set2:NCP complex

a) Fourier shell correlation (FSC) curve showing the resolution of Set2:NCP cryo-EM map.

b) Local resolution estimate with ResMap for Set2:NCP cryo EM map. Histone core and Set2 region that is proximal to the NCP are resolved to 3.5-3.9 Å. Set2 regions that are distal from the NCP have lower resolution, ranging from 4.0-5.0 Å.

c) The models of the NCP (PDB:6FQ5) and Set2 (PDB:5V21) were refined into cryo-EM map. Several representative regions showing fit of the model and local resolution are shown. NCP is shown in grey and Set2 in pink.

d) Schematic representation of Set2 domain organization.

e) Set2 residue R117 binds the backbone of the unwrapped DNA at SHL 5.5. NCP cryo-EM map and model are shown in gray and Set2 in pink.

f) Positively charged residue R117 in the loop interacting with the unwrapped DNA is highly conserved.

g) Set2 residues K241, K322 and T323 bind the backbone of the DNA at SHL -1. NCP cryo-EM map and model are shown in gray and Set2 in pink.

h) Set2 residues K322 and T323 bind the DNA and rearrange the Post-Set domain when compared to the crystal structure of Set2 in complex with the H3 peptide (PDB:5V21, 5JJY). Post-Set domain is partially delocalized in our structure.

i) Several residues in the H3 αN (K56, R52, R49) change their conformation after Set2 binding. The H3 αN in the NCP is show in gray and in the Set2:NCP complex in pink.

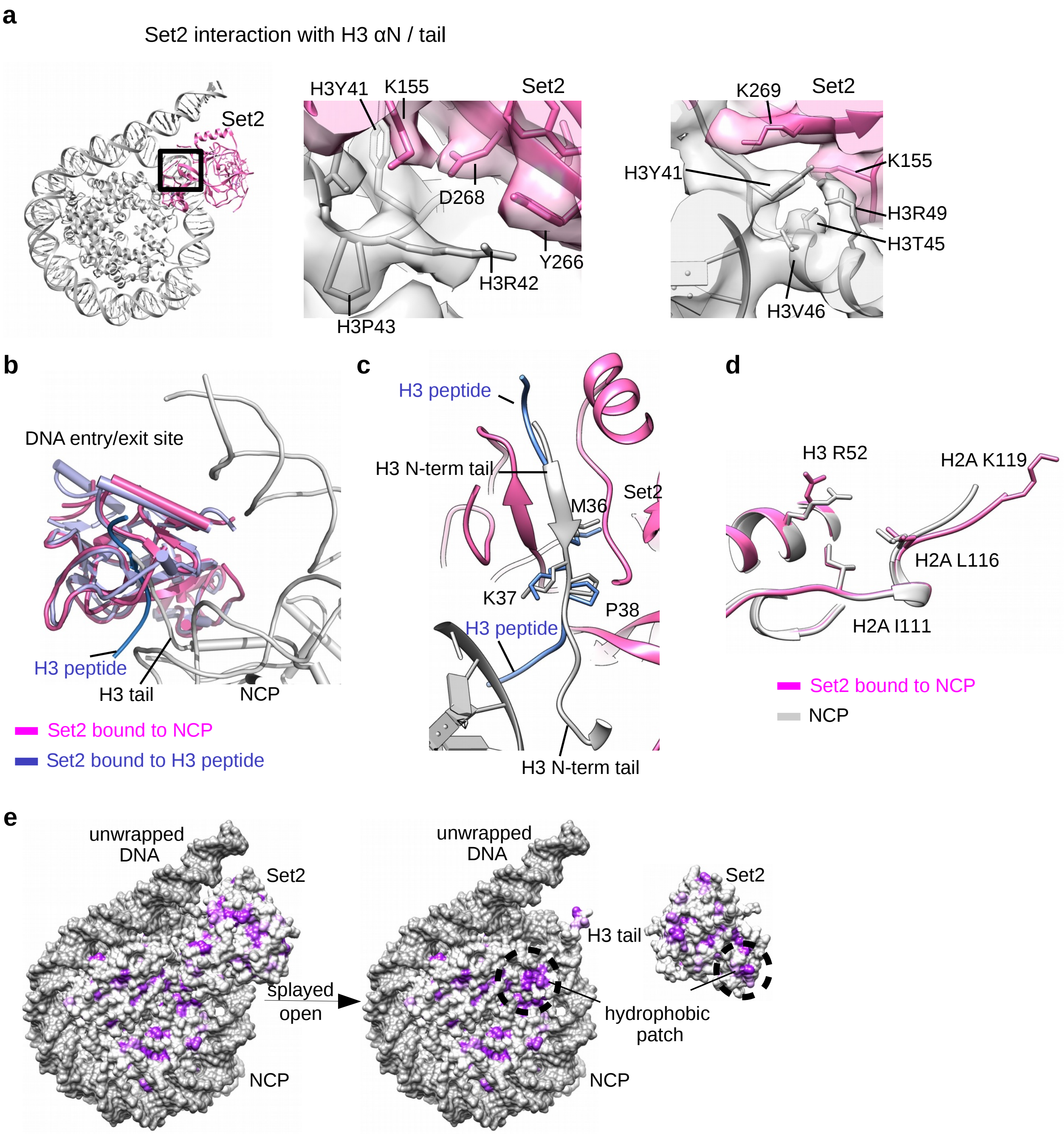

Supplementary Figure 4

Supplementary Fig. 4. Set2 interactions with NCP

a) Cryo-EM map and the model showing interaction between Set2 and the histone H3 tail.

b) Set2 crystal structure bound to H3 peptide (PDB:5V21, 5JJY) is overlayed with Set2 bound to the NCP. Note that H3 peptide takes a different path than the H3 tail in the cryo-EM structure.

c) Close up view of **b)**. H3 peptide in Set2 crystal structure (PDB:5V21, 5JJY) takes a different path than H3 tail in cryo-EM structure. The H3 tail path changes at P38.

d) Conformational change in H2A C-terminal tail after Set2 binding. The H3 αN in the NCP is show in gray and in the Set2:NCP complex in pink.

e) Hydrophobic residues on the surface of NCP and Set2 are shown in violet. Black dashed circles mark interacting hydrophobic patches on the NCP and Set2.

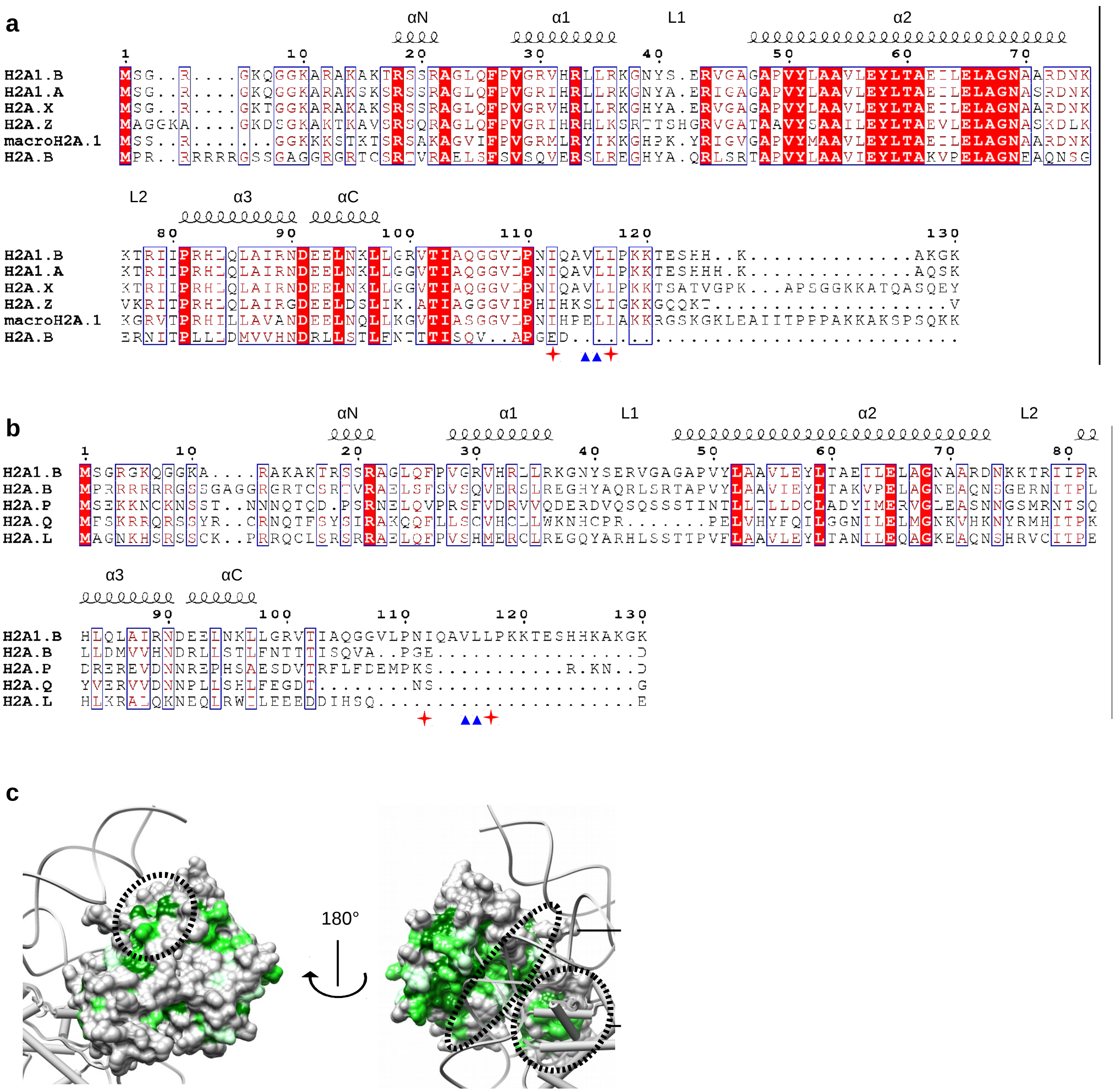

Supplementary Figure 5

Supplementary Fig. 5. H2A hydrophobic patch is not conserved in H2A.B variant

a) Sequence alignment of the representative H2A histone variants. Conserved residues are highlighted in red. The position of Ile111 and Leu116, residues that build hydrophobic pocket on the nucleosomal surface, are labeled with the red cross. Val 114 and Leu115 that orient H2A C-terminal tail are marked with the blue triangle. Residues important for the interactions with Set2 enzyme are not conserved in H2A.B.

b) Sequence alignment of human canonical and short H2A variants: H2A.B, H2A.P, H2A.Q, H2A.L. These, mammalian specific, H2A variants have shorter H2A C-terminus and thus no residues crucial for the interaction with H3K36 methylation enzymes. Residues directly interacting with Set2, Ile111 and Leu116, are labeled with the red cross. Residues important for proper positioning H2A C-terminus are labeled as blue triangle.

c) Conserved residues in Set2 are shown in green. Residues interacting with the nucleosome, marked with black dashed circles, show higher conservation. An additional conserved patch is not interacting with the nucleosome, indicating that this surface interacts with another binding partner.

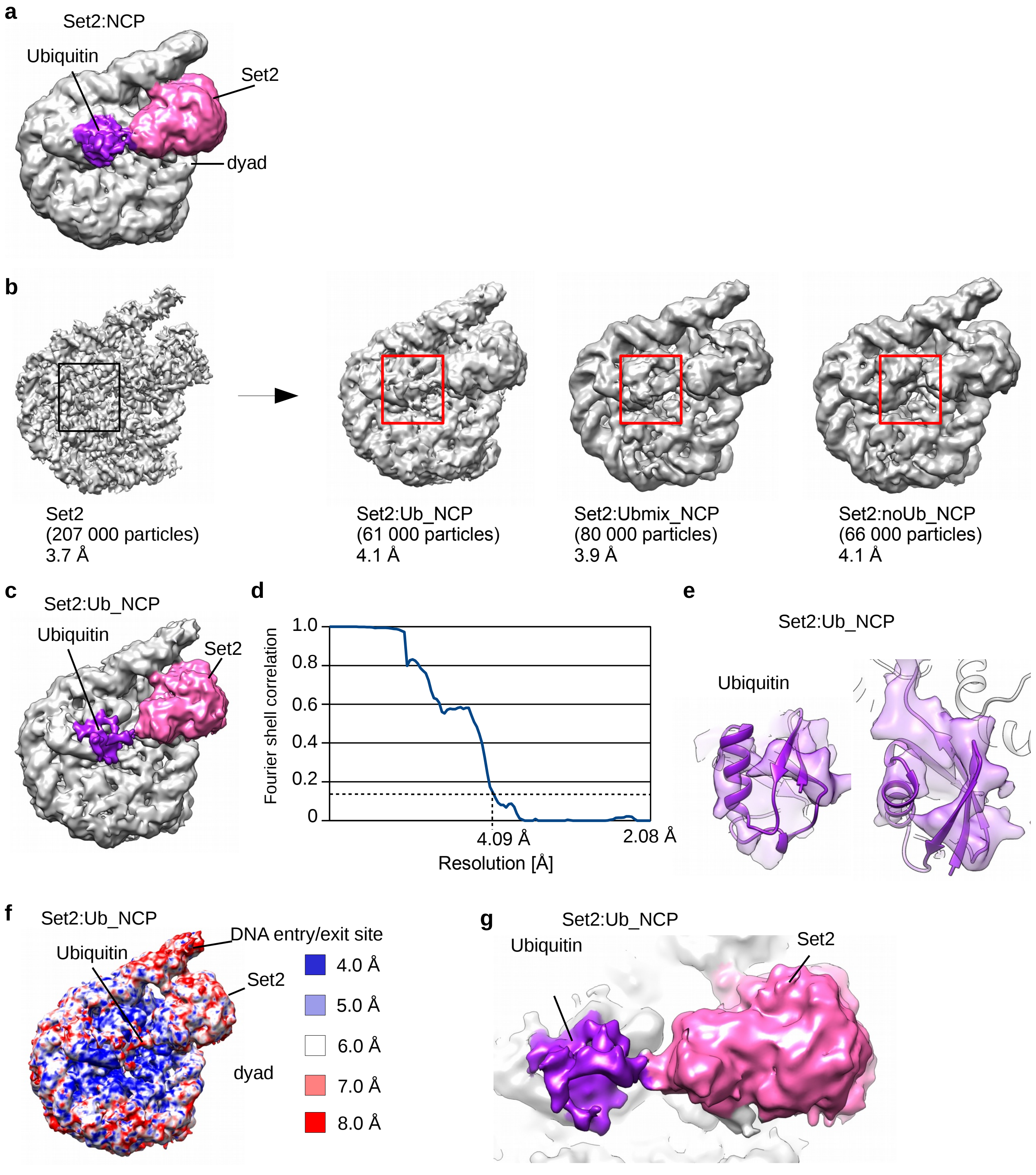

Supplementary Figure 6

Supplementary Fig. 6. Ubiquitin binds Set2 and stimulates Set2 H3K36me3 activity

a) Cryo-EM map of Set2:NCP is shown at lower contour level. At lower contour level, the density for ubiquitin is visible. Ubiquitin density is shown in violet, Set2 in pink and NCP in gray.

b) Set2:NCP was further classified to improve ubiquitin density. 3 classes are shown. Set2_Ub_NCP has defined density for ubiquitin; Set2_Ubmix_NCP has ubiquitin present in multiple conformations; Set2_noUb_NCP has visible density for ubiquitin, indicating that ubiquitin is delocalized.

c) Set2_Ub_NCP cryo-EM with improved ubiquitin density at 4.1 Å. Ubiquitin density is shown in violet, Set2 in pink and NCP in gray.

d) Fourier shell correlation (FSC) curve showing the resolution of Set2_Ub_NCP cryo-EM map.

e) The X-ray structure of ubiquitin (PDB:1UBQ) was docked into the Set2_Ub_NCP cryo-EM map. Ubiquitin α-helix and β-sheet are resolved in the ubiquitin density. Ubiquitin density is shown in transparent purple.

f) Local resolution estimate with ResMap for Set2_Ub_NCP cryo-EM map. Ubiquitin is resolved to 5-6 Å on the NCP proximal side and 6-8 Å on the distal side.

g) Set2_Ub_NCP cryo-EM density shows interaction between Set2 and ubiquitin.

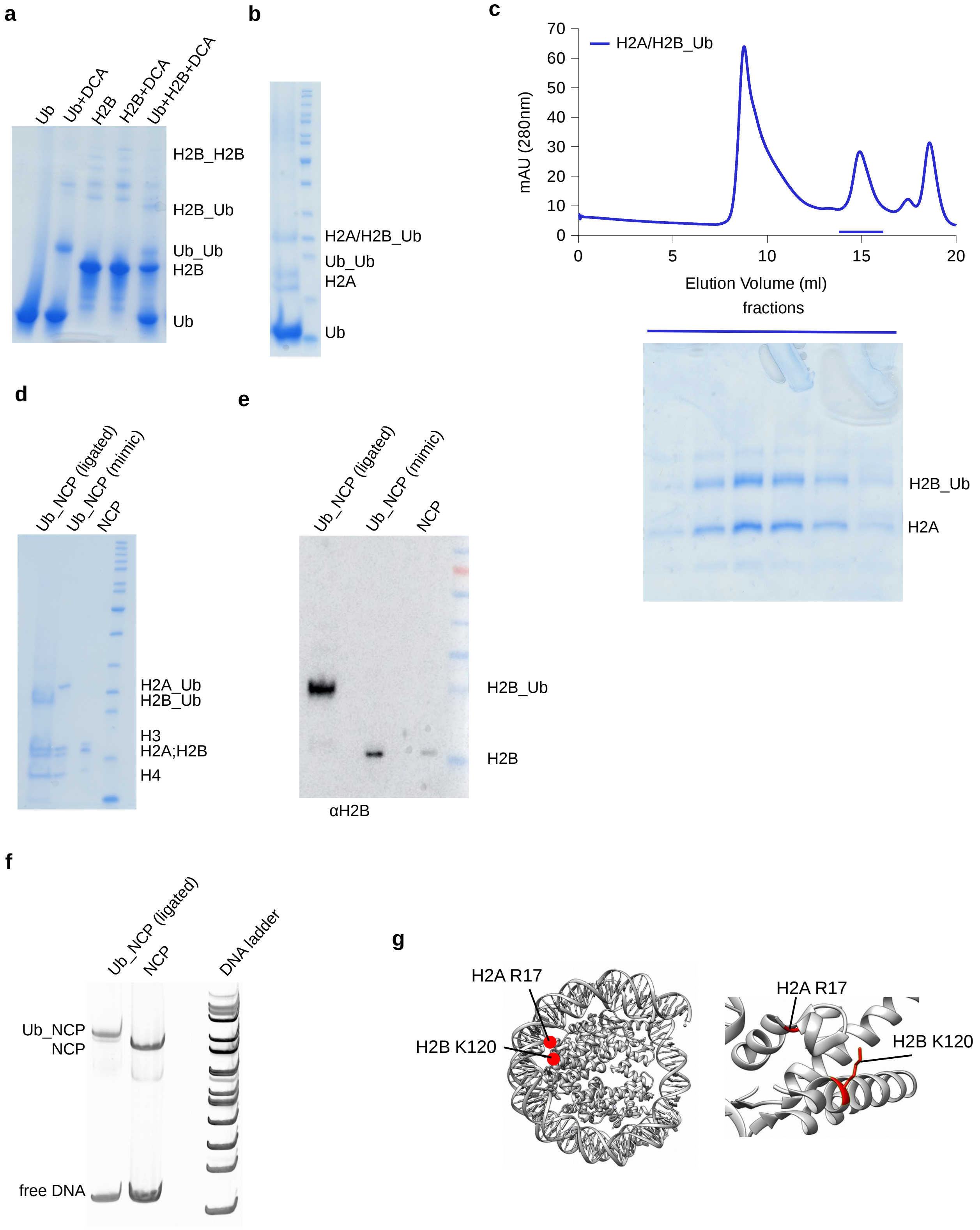

Supplementary Figure 7

Supplementary Fig. 7. Generation of H2BK120C ubiquitinated NCPs

a) SDS-PAGE showing cross-linking reactions of ubiquitin and H2BK120C histone.

b) Cross-linked ubiquitin–H2B histone mimic was refolded with H2A and the dimer was purified using Ni-NTA affinity resin. SDS-PAGE showing the elution fraction of the affinity resin purification.

c) Size exclusion chromatography profile of H2A/H2B_Ub dimer is shown in blue. Selected fractions, marked with a blue bar on the chromatogram, were analyzed by SDS-PAGE.

d) SDS-PAGE showing the NCP with H2BK120C ubiquitin in the first well, NCP with H2A fused to ubiquitin (second well) and the wild type NCP (third well).

e) Western blot using antibodies against H2B histone. H2BK120C ubiquitinated H2B migrates at higher molecular weight when compared to unmodified H2B.

f) Native gel showing the assembly of the H2BK120C ubiquitinated nucleosomes and the unmodified nucleosomes.

g) Location of H2BK120, naturally occurring ubiquitination residue, and H2AR17, ubiquitin ligation mimic position. The distance between two residues is ~10 Å.

Source data are provided as a Source Data file.

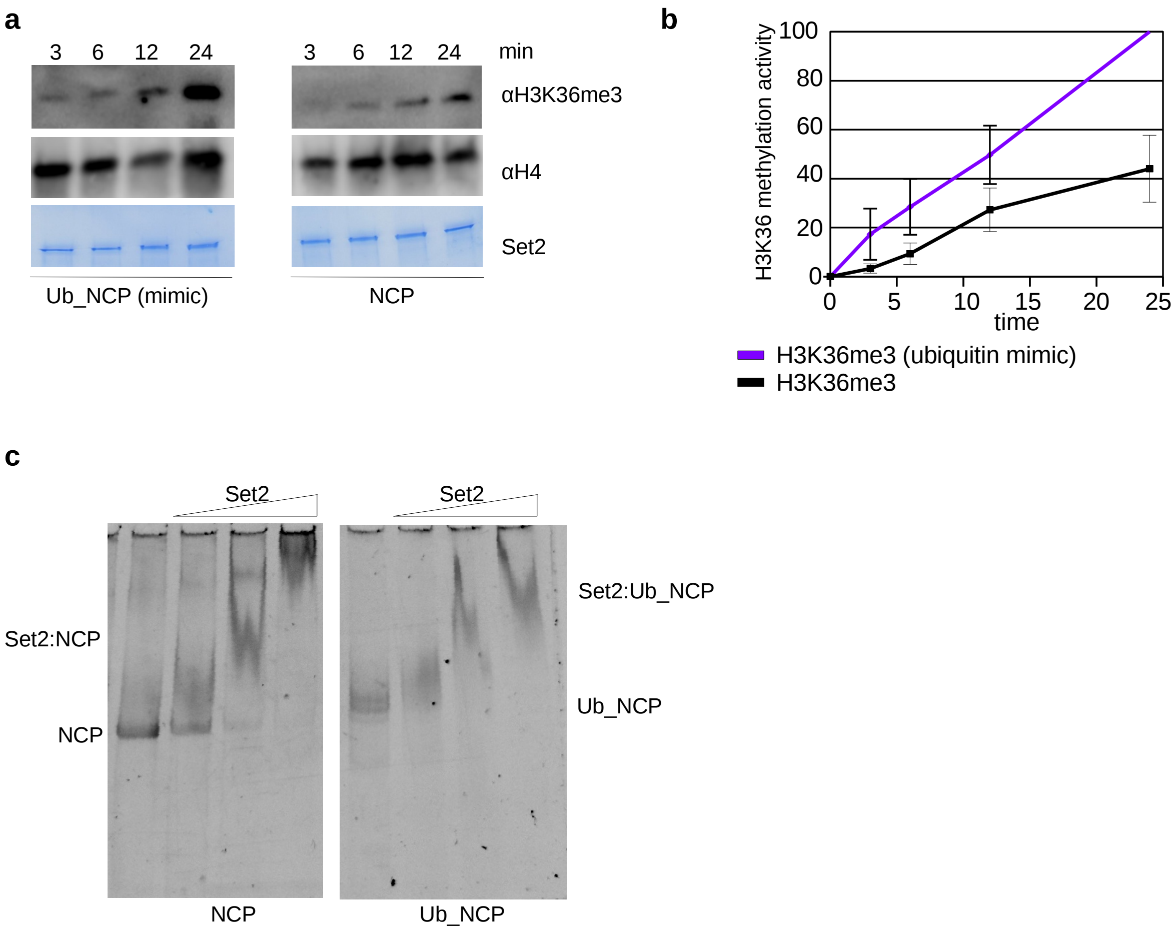

Supplementary Figure 8

Supplementary Fig. 8. Ubiquitin binds Set2 and stimulates Set2 H3K36me3 activity

a) Set2 methyltransferase assay with the unmodified and the H2B ubiquitin mimic nucleosomes. In presence of H2B ubiquitin mimic, Set2 shows increased H3K36me3 when compared to the unmodified nucleosome.

b) Quantification of Set2 methyltransferase activity assay from **a)**. Error bars show standard error of four independent experiments.

c) Native gel showing Set2 binding to unmodified and H2BK120C ubiquitinated nucleosomes.

Source data are provided as a Source Data file.

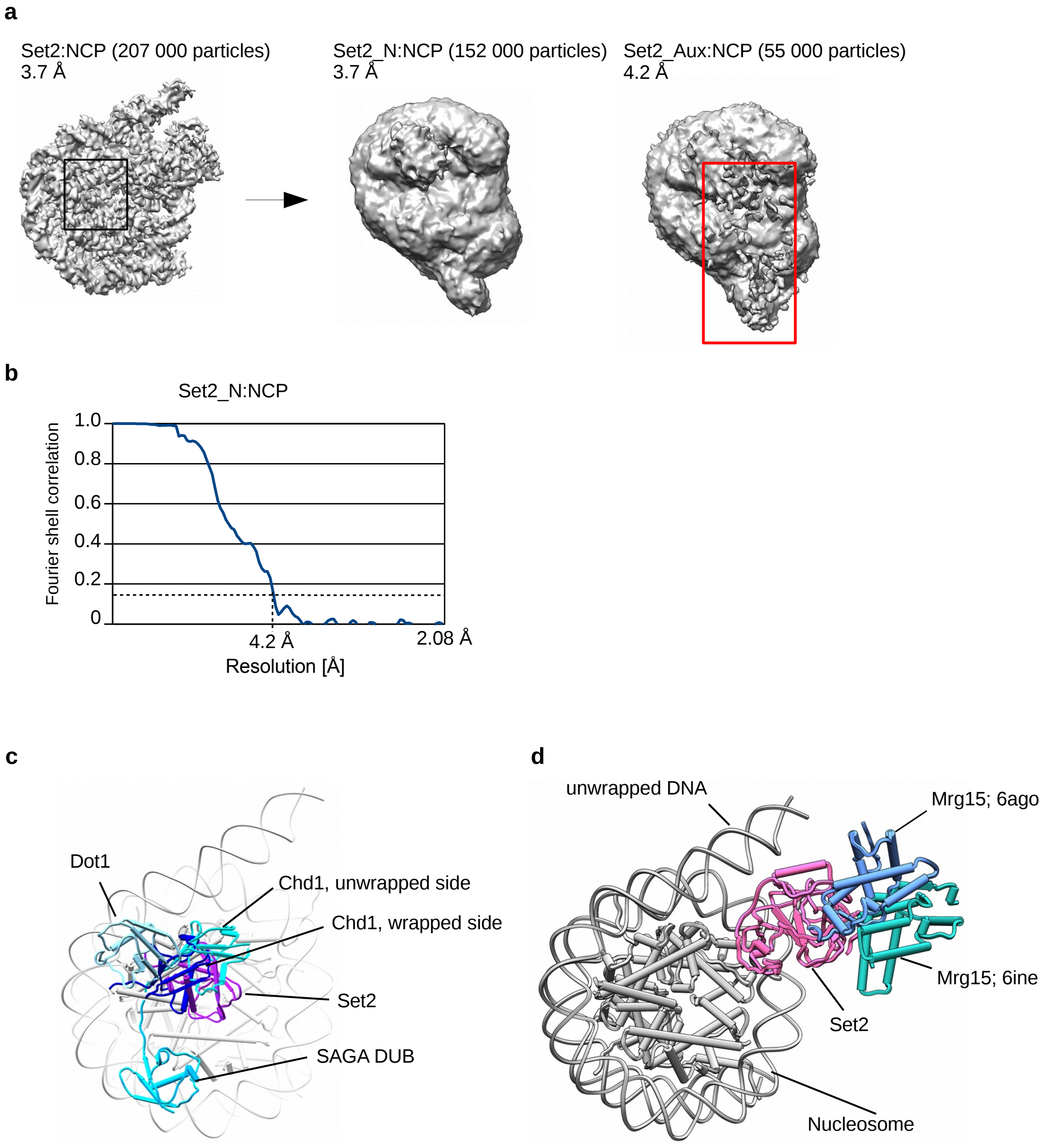

Supplementary Figure 9

Supplementary Fig. 9. Set2 auxiliary domains bind the nucleosome.

a) Set2:NCP was further classified which leads to appearance of the class with the noisy density spanning from the Set2 SET domain to the ubiquitin and H2A/H2B on the opposite side of the nucleosome. This class, Set2_Aux:NCP contains ~25% of particles. Neither mask nor focus classification were used to obtain this class.

b) Fourier shell correlation (FSC) curve showing the resolution of Set2_Aux:NCP cryo-EM map.

c) The position of H2BK120 ubiquitin is influenced by nucleosome binding factors. Global location of the ubiquitin on the nucleosome in different structures: Set2 (violet), SAGA DUB module (sky blue), Dot1 (light blue), Chd1 (unwrapped DNA side in cyan, wrapped DNA side in dark blue). NCP from Set2:NCP complex structure is shown in gray.

d) The Set2:NCP cryo-EM model superimposed on the ASH1L:MRG15 crystal structures (PDB ID: 6ago, 6ine). SET domains from ASH1L complexes are omitted for clarity. MRG15 in two X-ray structures have different orientation with respect to SET domain. Set2 is shown in pink, NCP in gray, MRG15 from PDBID:6ago in blue and MRG15 from PDBID:6ine in teal.

Supplementary Table 1: List of oligonucleotides used in the study

Oligonucleotides	Seguence
149 bp 601 DNA	
423F	GCA CAG GAT GTA TAT ATC TG
423R	CTG GAG AAT CCC GGT
Set2 cloning into pET-Duet	
854F	ATCT GGATCC ATGGAGGCAAGTGACCGT
854R	GCA TGA ATT CTC AAT GGC TCA TCA CAG CTT G
Set2 intron1 removal	
856F	GCG ACG GCA AGA ACC AC
856R	GCC AAT CCT CAG CAC AAT C
Set2 intron2 removal	
857F	CTC TTT CCT CAC GTC AAG
857R	TGT GTT CTC ATA GAT CTT CAT
H3K36M mutagenesis	
1002F	TGA AAC CTC ACC GTT ACC GGC
1002R	TGA CTC CGC CGG TAG CAG
Ubq + H2A/H2B	
916F	TATTCCATGGGTATGCAGATTTTCGTCAAGAC
916R	TATACCATGGACCTCTTAGCCTTAGCACAAG
Ubq_H2A/H2B	
941F	CGGCGGCAGCGGCGGCTCAGGTCGCTCATCTCG
941R	CTGCCGCCGCTGCTAGTACCTCTTAGCCTTAGC
H2BK120C mutagenesis	
960F	TGT TAC ACC AGC GCC AAG TAA
960R	GGT GAC AGC CTT GGT GC

Supplementary Table 2: Plasmids used in this study

298	pET3_Widom 601 DNA
409	pET3_H4
889	pET29_ 6xHis_HRV3C_H2A/H2B
905	pETDuet_ 6xHis_HRV3C_Set2
1093	pET29_ 6xHis_Ubq_H2A/H2B
1201	pET29_ 6xHis_HRV3C_H3/H4
1213	pET3_H3K36M
971	pET3_H2BK120C
