# Supplementary material for: Nucleosome and ubiquitin position Set2 to methylate H3K36
Source: Nat Commun. 2019 Aug 22;10:3795. doi: 10.1038/s41467-019-11726-4 (PMC6706414; doi:10.1038/s41467-019-11726-4)
Supplement: Supplementary file 4 — Source Data [file 41467_2019_11726_MOESM4_ESM.pdf]

Fig. 4c

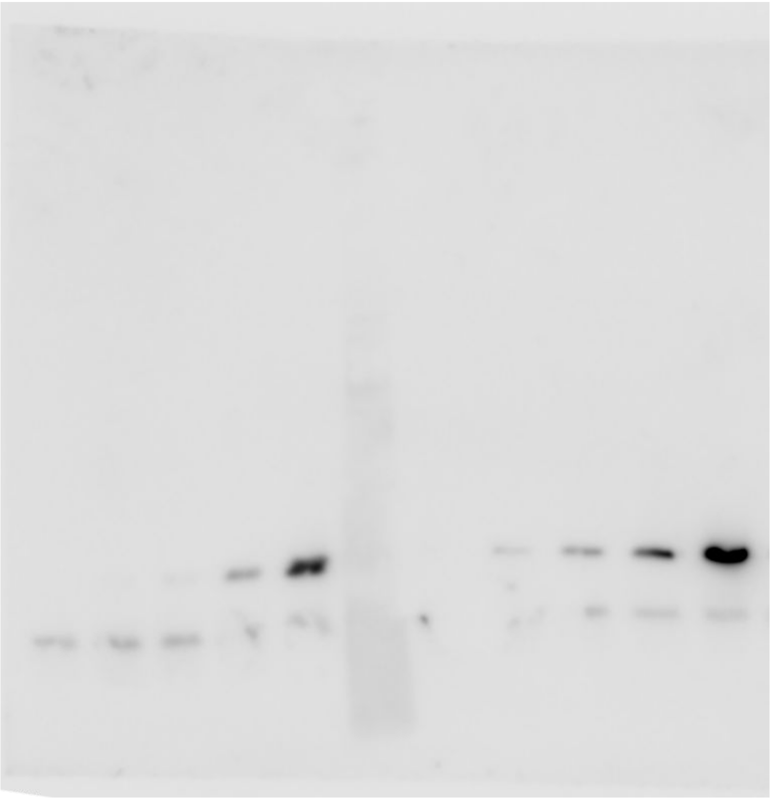

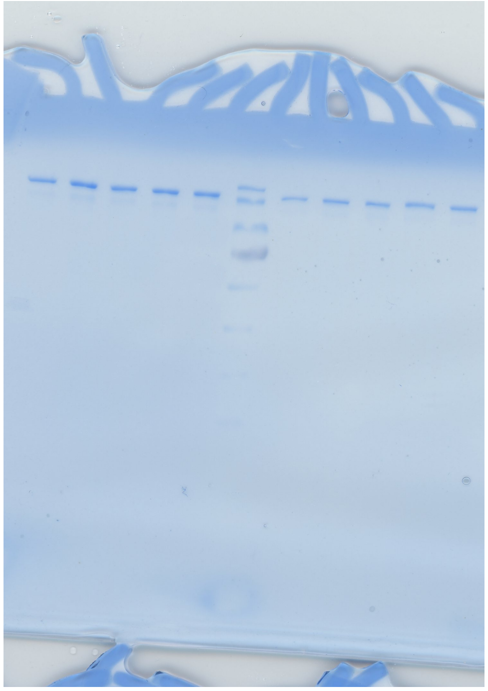

Fig. 4c

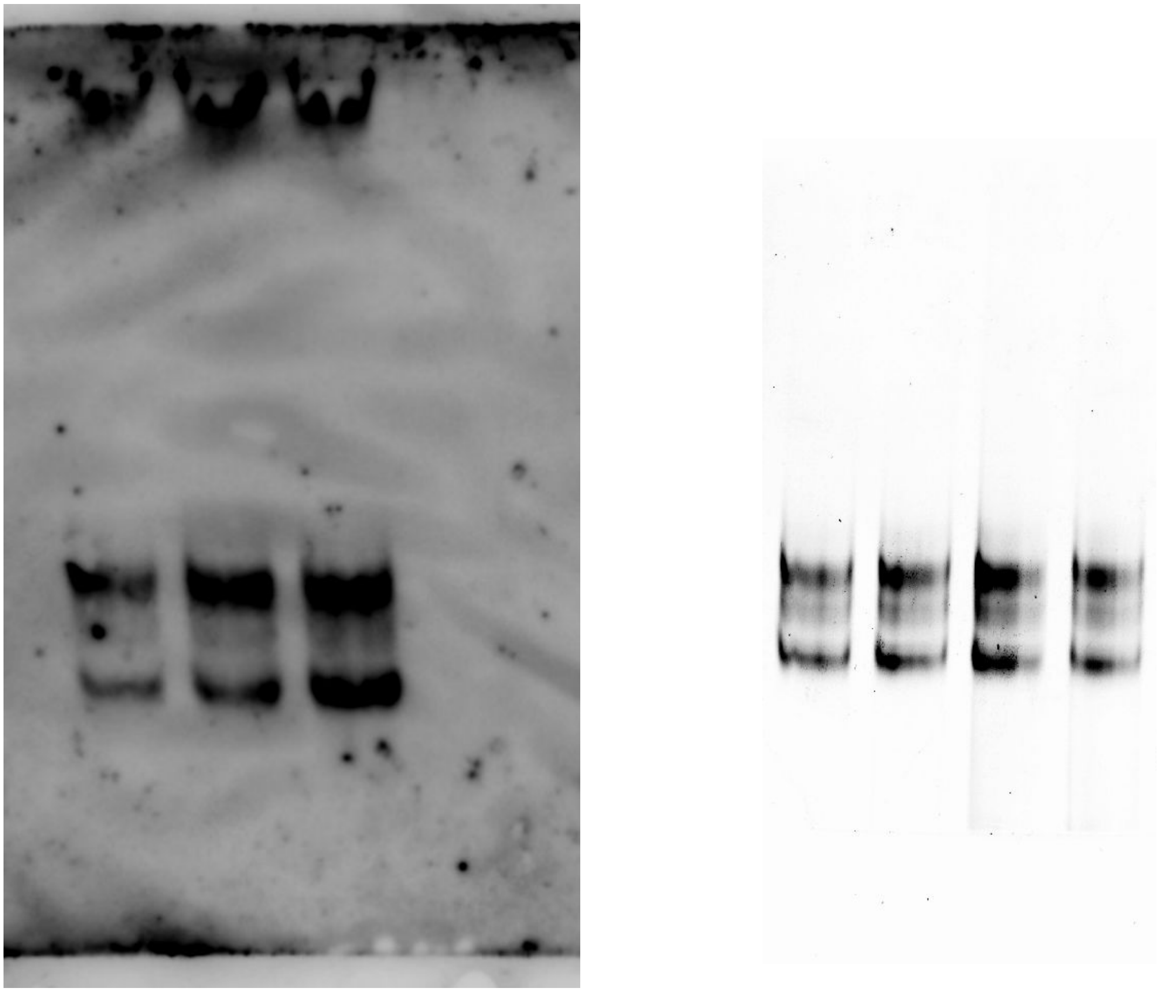

Fig. 4e

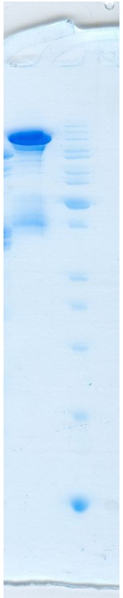

Supplementary Fig. 1a

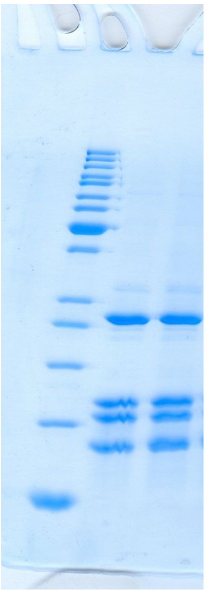

Supplementary Fig. 1b

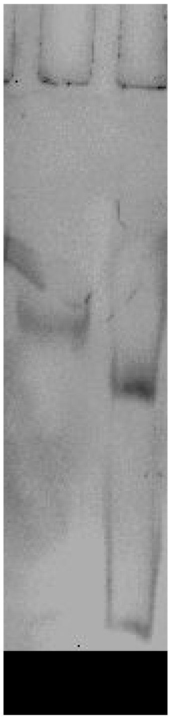

Supplementary Fig. 1c

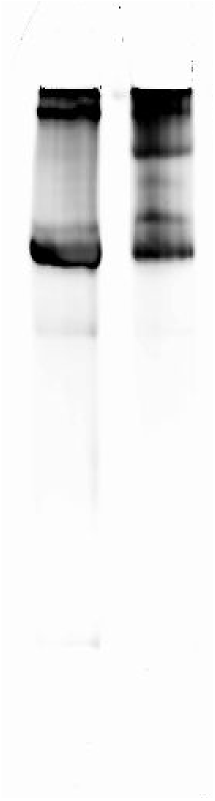

Supplementary Fig. 1d

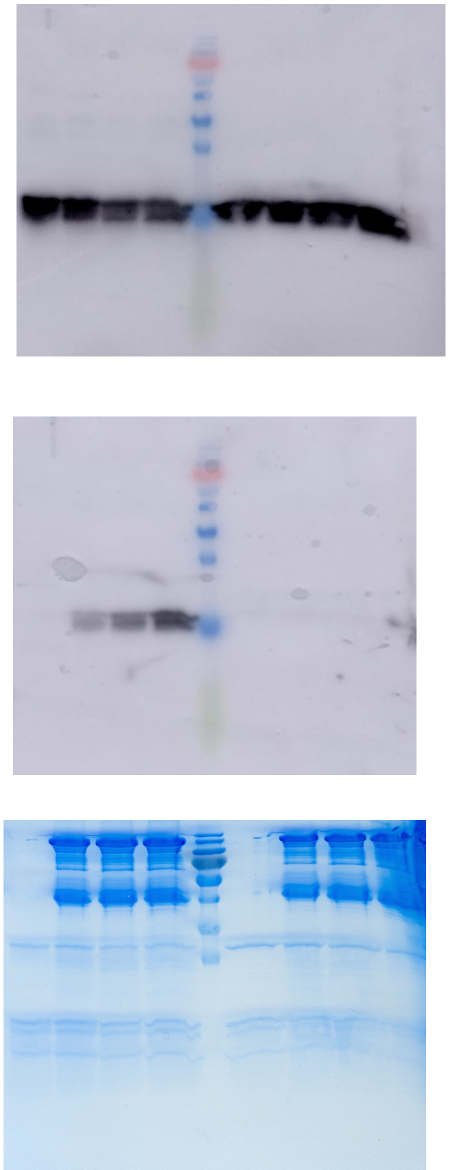

Supplementary Fig. 1e

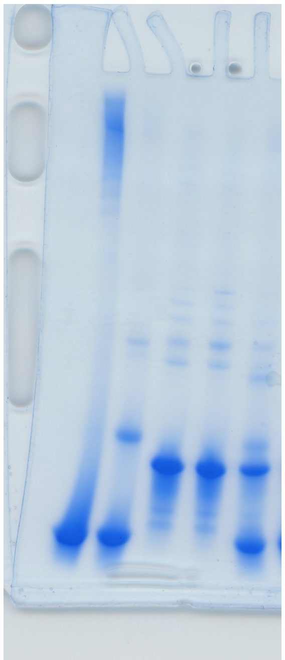

Supplementary Fig. 7a

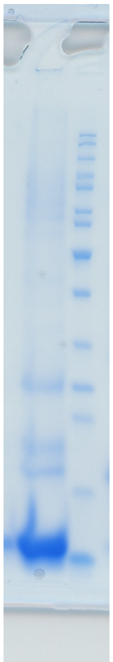

Supplementary Fig. 7b

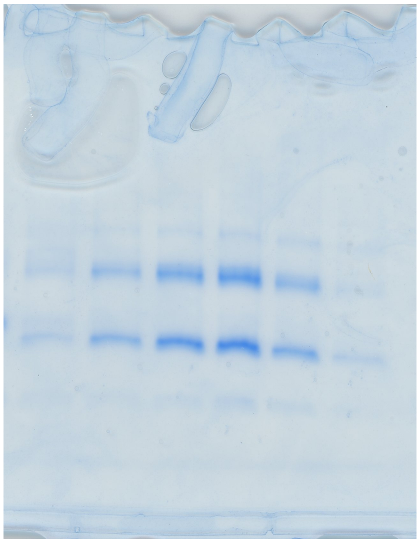

Supplementary Fig. 7c

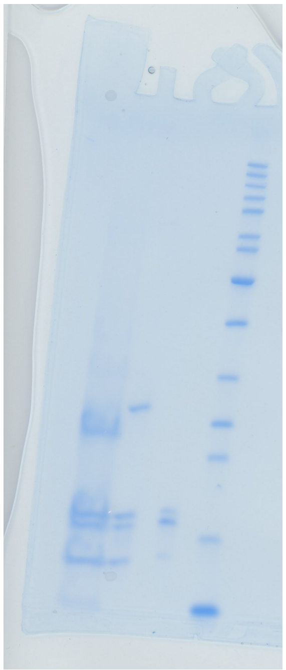

Supplementary Fig. 7d

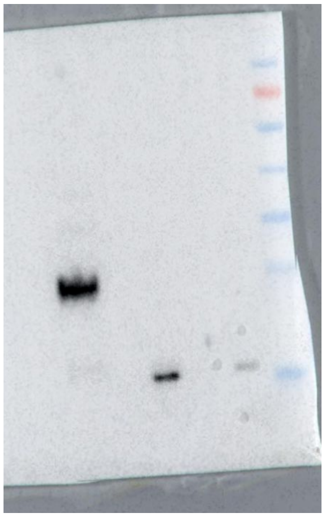

Supplementary Fig. 7e

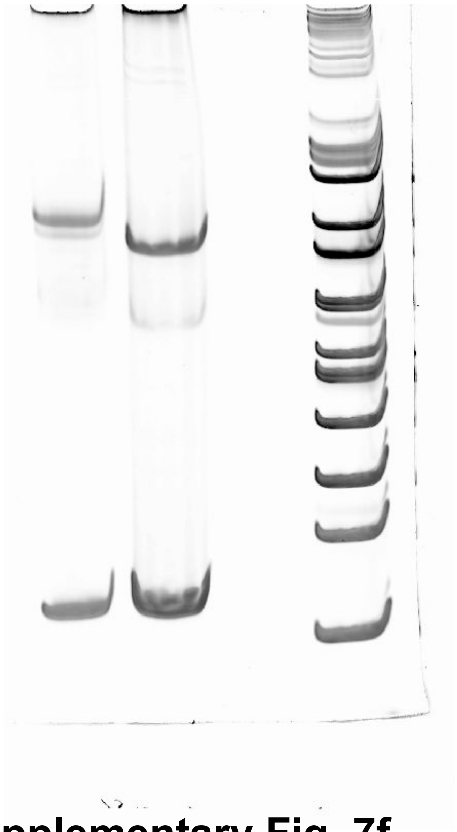

Supplementary Fig. 7f

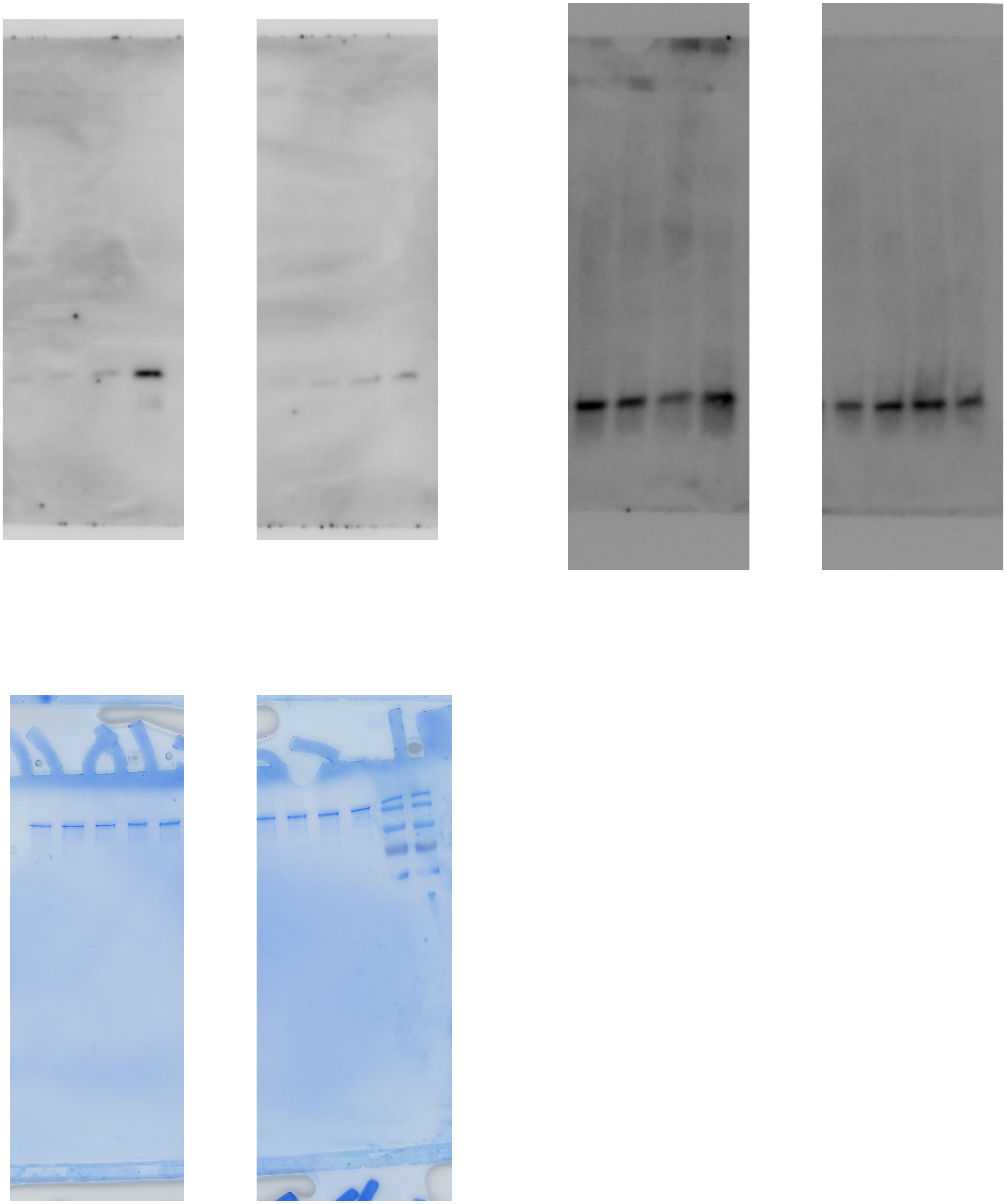

Supplementary Fig. 8a

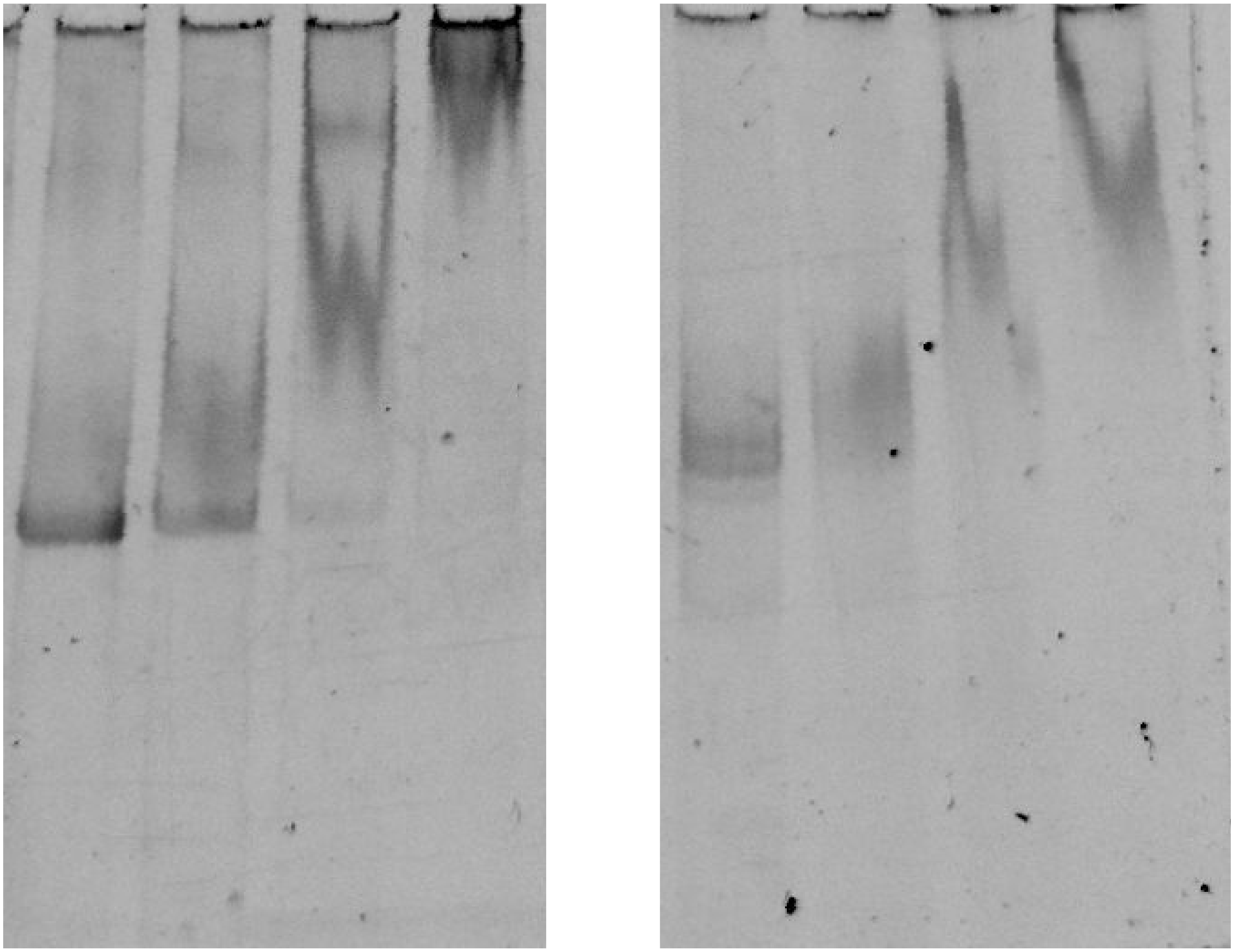

Supplementary Fig. 8c
