# Supplementary material for: Nucleosome and ubiquitin position Set2 to methylate H3K36
Source: Nat Commun. 2019 Aug 22;10:3795. doi: 10.1038/s41467-019-11726-4 (PMC6706414; doi:10.1038/s41467-019-11726-4)
Supplement: Supplementary file 2 — Peer Review File [file 41467_2019_11726_MOESM2_ESM.pdf]

Editorial Note: This manuscript has been previously reviewed at another journal that is not operating a transparent peer review scheme. The manuscript was considered suitable for publication without further review at *Nature Communications*.
